# Interpretation of 10 years of Alzheimer’s disease genetic findings in the perspective of statistical heterogeneity

**DOI:** 10.1093/bib/bbae140

**Published:** 2024-05-06

**Authors:** Shan Gao, Tao Wang, Zhifa Han, Yang Hu, Ping Zhu, Yanli Xue, Chen Huang, Yan Chen, Guiyou Liu

**Affiliations:** Beijing Institute of Brain Disorders, Laboratory of Brain Disorders, Ministry of Science and Technology, Collaborative Innovation Center for Brain Disorders, Capital Medical University, No. 10, Xitoutiao, You’an Men Wai, Fengtai District, Beijing 100069, China; Chinese Institute for Brain Research, No. 26, Kexueyuan Road, Changping District, Beijing 102206, China; State Key Laboratory of Medical Molecular Biology, Institute of Basic Medical Sciences, Chinese Academy of Medical Sciences, No. 5, Dongdan Santichao, Dongcheng District, Beijing 100193, China; School of Computer Science and Technology, Harbin Institute of Technology, No. 92, Xidazhi Street, Nangang District, Harbin 150006, China; Beijing Institute of Brain Disorders, Laboratory of Brain Disorders, Ministry of Science and Technology, Collaborative Innovation Center for Brain Disorders, Capital Medical University, No. 10, Xitoutiao, You’an Men Wai, Fengtai District, Beijing 100069, China; School of Biomedical Engineering, Capital Medical University, No. 10 Xitoutiao, You'an Men Wai, Fengtai District, Beijing 100069, China; Dr. Neher’s Biophysics Laboratory for Innovative Drug Discovery, State Key Laboratory of Quality Research in Chinese Medicine, Macau University of Science and Technology, Avenida WaiLong, Taipa 999078, Macao SAR, China; Department of Epidemiology and Biostatistics, School of Public Health, Wannan Medical College, No. 22, Wenchang Road, Wuhu 241002, Anhui, China; Institute of Chronic Disease Prevention and Control, Wannan Medical College, No. 22, Wenchang Road, Wuhu 241002, Anhui, China; Chinese Institute for Brain Research, No. 26, Kexueyuan Road, Changping District, Beijing 102206, China; Department of Epidemiology and Biostatistics, School of Public Health, Wannan Medical College, No. 22, Wenchang Road, Wuhu 241002, Anhui, China; Institute of Chronic Disease Prevention and Control, Wannan Medical College, No. 22, Wenchang Road, Wuhu 241002, Anhui, China; Key Laboratory of Cerebral Microcirculation in Universities of Shandong, Department of Neurology, Second Affiliated Hospital, Shandong First Medical University and Shandong Academy of Medical Sciences, Taian 271000, Shandong, China; Beijing Key Laboratory of Hypoxia Translational Medicine, National Engineering Laboratory of Internet Medical Diagnosis and Treatment Technology, Xuanwu Hospital, Capital Medical University, No. 45, Changchun Road, Xicheng District, Beijing 100053, China

**Keywords:** Alzheimer’s disease, genome-wide association studies, GWAS by proxy, statistical heterogeneity, phenotypic heterogeneity

## Abstract

Common genetic variants and susceptibility loci associated with Alzheimer’s disease (AD) have been discovered through large-scale genome-wide association studies (GWAS), GWAS by proxy (GWAX) and meta-analysis of GWAS and GWAX (GWAS+GWAX). However, due to the very low repeatability of AD susceptibility loci and the low heritability of AD, these AD genetic findings have been questioned. We summarize AD genetic findings from the past 10 years and provide a new interpretation of these findings in the context of statistical heterogeneity. We discovered that only 17% of AD risk loci demonstrated reproducibility with a genome-wide significance of *P* < 5.00E-08 across all AD GWAS and GWAS+GWAX datasets. We highlighted that the AD GWAS+GWAX with the largest sample size failed to identify the most significant signals, the maximum number of genome-wide significant genetic variants or maximum heritability. Additionally, we identified widespread statistical heterogeneity in AD GWAS+GWAX datasets, but not in AD GWAS datasets. We consider that statistical heterogeneity may have attenuated the statistical power in AD GWAS+GWAX and may contribute to explaining the low repeatability (17%) of genome-wide significant AD susceptibility loci and the decreased AD heritability (40–2%) as the sample size increased. Importantly, evidence supports the idea that a decrease in statistical heterogeneity facilitates the identification of genome-wide significant genetic loci and contributes to an increase in AD heritability. Collectively, current AD GWAX and GWAS+GWAX findings should be meticulously assessed and warrant additional investigation, and AD GWAS+GWAX should employ multiple meta-analysis methods, such as random-effects inverse variance-weighted meta-analysis, which is designed specifically for statistical heterogeneity.

## INTRODUCTION

Alzheimer’s disease (AD), the most prevalent neurodegenerative disorder, arises from genetic factors, environmental factors and their interplay [1–7]. Until recently, the identification of the most common genetic variants and risk loci associated with AD has primarily relied on genome-wide association studies (GWAS) conducted in clinically diagnosed AD and cognitively normal controls [1–6, 8]. Since 2017, GWAS for family history of AD, known as GWAS by disease proxy (GWAX), has gradually been proposed as a powerful method to increase the sample size using UK Biobank participants [9–13]. Meanwhile, AD GWAS and GWAX datasets have been further integrated through a meta-analysis (GWAS+GWAX) and highlighted some additional novel findings [10–16].

However, recent GWAS+GWAX studies have cast doubt on the genetic findings related to AD within the scope of common variants due to their low repeatability in identifying AD susceptibility loci and the low heritability of AD [16–18]. First, only a limited proportion, approximately 37.5%, of the AD susceptibility loci could be recreated with genome-wide significance (*P* < 5.00E-08) across the AD GWAS and GWAS+GWAX datasets [18]. Crucially, despite utilizing the largest sample size, GWAS+GWAX was unable to identify the most significant signals for the majority of the known AD loci, nor did it identify the maximum number of genome-wide significant AD loci [14, 15]. The exponential increase in sample sizes only identified a marginally increased number of novel AD susceptibility loci [17]. Second, the heritability explained by these AD GWAS and GWAS+GWAX datasets decreased from 40 to 2% as the overall sample size, especially the GWAX sample size, increased [17]. A subgroup analysis indicated that the SNP-based heritability on a liability scale (with a 5% prevalence) decreased from 6% (s.e. = 0.01) excluding the UK Biobank cohort to 2% (s.e. = 0.003) including the UK Biobank cohort [11, 17]. Counterintuitively, it seems that bigger is not always better [17].

Here, we reconsider recent 10 years of AD genetic findings and provide a new interpretation of these findings in the context of statistical heterogeneity. We searched PubMed for AD GWAS and GWAS+GWAX published between 1 April 2009 and 1 April 2023, focusing on individuals of European descent and using the following keywords: ‘Alzheimer’s disease’, ‘genome-wide association studies’, ‘GWAS’, ‘GWAS by proxy’ and ‘GWAX’. The final bibliography was created considering the relevance of the subjects discussed in this review. In stage 1, we summarize recent AD GWAS and GWAS+GWAX findings and evaluate their repeatability. In stage 2, we emphasize statistical heterogeneity and interpret the findings from AD GWAS and GWAS+GWAX within the context of statistical heterogeneity. In stage 3, we discuss the potential sources of statistical heterogeneity in the findings of AD GWAS and GWAS+GWAX.

## AD GENETIC FINDINGS

### AD GWAS findings

GWAS is a major and popular method to identify the common genetic variants associated with them at the genome-wide significance level (*P* < 5.00E-08) [18]. Until now, AD GWAS have demonstrated enhanced statistical power by incorporating clinically or pathologically diagnosed AD and healthy controls [18]. AD GWAS with an increased sample size not only confirms previous GWAS findings but also highlights some novel findings [1–6, 8]. In 2009, Lambert *et al.* [3] conducted a two-stage AD GWAS: discovery stage with 2032 AD and 5328 controls, and replication stage with 3978 AD cases and 3297 controls. A meta-analysis of both stages highlighted two AD loci, *CLU* and *CR1* [3]. Harold *et al.* [5] identified *CLU* and *PICALM* to be the AD susceptibility loci using 3941 cases and 7848 controls in the discovery stage, and 2023 cases and 2340 controls in the replication stage. In 2010, Seshadri *et al.* [1] further added *BIN1* and *EXOC3L2/BLOC1S3/MARK4* as novel AD susceptibility loci using a large-scale sample size. In 2011, Hollingworth *et al.* [2] undertook an extensive meta-analysis of GWAS, analyzing a total of 19 870 AD cases and 39 846 controls. They highlighted five novel AD susceptibility loci, including *CD2AP*, *ABCA7*, *EPHA1*, *MS4A6A/MS4A4E* and *CD33* [2]. Importantly, they further confirmed *CR1* and *BIN1* [2]. Naj *et al.* [6] performed a large-scale GWAS using a three-stage design, including stage 1 (8309 AD and 7366 controls), stages 2 (3531 AD and 3565 controls) and stage 3 (7650 AD and 25 839 controls). They identified *CD33*, *CD2AP*, *MS4A4/MS4A6E* and *EPHA1* to be novel AD susceptibility genes [6]. Importantly, they further confirmed *CR1*, *CLU*, *BIN1* and *PICALM*, but not *EXOC3L2*, to be the AD risk loci [6]. Until 2013, the International Genomics of Alzheimer’s Project (IGAP) conducted the most extensive meta-analysis of GWAS for AD, encompassing a sample size of 74 046 individuals of European ancestry, including 25 580 cases of AD and 48 466 controls [4]. In addition to *APOE*, IGAP 2013 identified 19 AD susceptibility loci, including 11 novel loci (*SORL1*, *FERMT2*, *SLC24A4/RIN3*, *PTK2B*, *ZCWPW1*, *HLA-DRB5–HLA-DRB1*, *MEF2C*, *INPP5D*, *CELF1*, *NME8* and *CASS4*) and 8 known loci (*MS4A6A*, *CR1*, *CD2AP*, *CD33*, *PICALM*, *EPHA1*, *CLU*, *ABCA7* and *BIN1*) [4]. In 2019, IGAP conducted the largest AD GWAS in 94 437 (35 274 individuals clinically diagnosed with AD and 59 163 controls) individuals of European descent from stage 1 (*n* = 63 926), stage 2 (*n* = 18 845) and stage 3A (*n* = 11 666) or stage 3B (*n* = 30 511) (GWAS2019) [8]. Finally, GWAS2019 identified 26 AD susceptibility loci, including 7 novel loci (*IQCK*, *ACE*, *ADAM10*, *ADAMTS1*, *WWOX*, *ECHDC3* and *OARD1*) and 19 previously known loci (18 loci were identified by IGAP 2013 excluding *TREM2)* [8]. Supplementary Tables SS1 and SS2 provide the detailed information about these 19 and 26 independent known AD genetic variants and susceptibility loci [4, 8].

### AD GWAX and GWAS+GWAX findings

In 2017, Liu *et al.* [9] first proposed GWAX to expand the sample size in genetic association investigations of complex traits by leveraging large population cohorts. GWAX overcomes the limitations from traditional GWAS, such as being costly and time-consuming, due to the substantial efforts required for recruitment, ascertainment and genotyping [9, 18]. It is reported that GWAX is more powerful when there are about four times (or more) as many proxy cases and controls as the traditional GWAS using true cases and controls [9, 18]. Liu *et al.* [9] applied the GWAX method to 12 common human diseases including AD in 116 196 individuals (14 482 proxy cases for AD and 100 082 controls) from the UK Biobank (GWAX2017). They further performed a meta-analysis combining the results from the IGAP 2013 AD GWAS and GWAX2017, and identified 25 genome-wide significant genetic variants corresponding to 22 AD susceptibility loci, including four novel AD susceptibility loci (*HBEGF*, *ECHDC3*, *SPPL2A* and *SCIMP*) [9].

Thereafter, AD GWAX and GWAS+GWAX were widely used to identify novel AD loci. In 2018, Marioni *et al.* [10] first conducted a GWAX using a dataset of 314 278 participants from the UK Biobank, which comprised 27 696 maternal AD cases and 14 338 AD paternal cases (GWAX2018). They identified six genome-wide significant loci, all situated within established AD loci, including *CR1*, *BIN1*, *PILRA*, *PICALM*, *ZNF232* and *APOE/TOMM40* [10]. They further meta-analyzed the AD GWAX and AD GWAS from IGAP 2013 (*n* = 74 046) and found 26 genome-wide significant genetic variants corresponding to 26 AD susceptibility loci including 19 known loci and 7 novel loci (*IL-34, SPPL2A, BCKDK/KAT8, ADAM10, TREML2, PLCG2, ACE*) (GWAS+GWAX2018) [10]. In 2019, Jansen *et al.* [11] conducted a large-scale GWAS meta-analysis using clinically diagnosed AD and controls from PGC-ALZ, IGAP 2013 and ADSP (*n* = 79 145, including 24 087 AD and 55 058 controls). They further conducted a GWAX using 376 113 individuals (47 793 proxy AD and 328 320 controls) from the UK Biobank (GWAX2019) [11]. Jansen *et al.* [11] discovered a robust genetic correlation between AD GWAS and GWAX and further performed a meta-analysis that integrated the findings from both AD GWAS and GWAX, involving a total of 455 258 individuals (GWAS+GWAX2019). Finally, Jansen *et al.* [11] identified 29 genome-wide significant genetic variants corresponding to 29 AD susceptibility loci, including nine novel loci (*AC074212.3*, *KAT8*, *CLNK*, *ALPK2*, *APH1B*, *CNTNAP2*, *ADAM10*, *ADAMTS4* and *HESX1*).

In 2021, three AD GWAS+GWAX have been reported [12–14]. Schwartzentruber *et al.* [12] first performed a GWAX using 53 042 proxy cases and 355 900 controls from UK Biobank (GWAX2021). They further conducted a meta-analysis of GWAX 2021 and IGAP 2019 stage 1 (21 982 AD and 41 944 controls) (GWAS+GWAX2021a) [12]. They identified 33 genome-wide significant genetic variants corresponding to 33 AD susceptibility loci, including four newly identified loci *TSPAN14*, *CCDC6*, *NCK2* and *SPRED2* [12]. Wightman *et al.* [14] performed a meta-analysis using 1 126 563 individuals from 18 cohorts, including IGAP, UK Biobank, GR@ACE, Finngen, DeCODE, 23andMe, HUNT, BioVU, TwinGene, DemGene1, DemGene2, DemGene3, DemGene4, DemGene5, STSA, ANMerge1, ANMerge23 and Gothenburg (GWAS+GWAX2021b). Wightman *et al.* identified 38 genome-wide significant genetic variants corresponding to 38 AD susceptibility loci, including seven newly discovered, namely *TNIP1*, *AGRN*, *NTN5*, *HAVCR2*, *TMEM106B*, *GRN* and *LILRB2* [14]. de Rojas *et al.* [19] conducted a large-scale meta-analysis of AD GWAS and GWAX by combining the discovery datasets from GR@ACE, IGAP 2019 stage 1 + 2 [8], UK Biobank [10] and the replication datasets from the European Alzheimer’s Disease Biobank (JPND-EADB) project, PGC-ALZ, GR@ACE, AD and GBCS, Neocodex and AddNeuroMed (GWAS+GWAX2021c) [13]. de Rojas *et al.* [13] identified 36 genome-wide significant genetic variants corresponding to 35 AD susceptibility loci, including five novel loci: *APP*, *CHRNE*, *PRKD3/NDUFAF7*, *PLCG2* and *SHARPIN*.

In 2022, Bellenguez *et al.* [15] performed a two-stage GWAS using 62 051 clinically diagnosed AD, 49 275 proxy AD and 677 663 controls (GWAS+GWAX2022a). They found 83 genome-wide significant genetic variants corresponding to 75 AD susceptibility loci, including 42 newly identified loci [15]. de la Fuente *et al.* [16] performed a meta-analysis of IGAP 2019 stage 1 (21 982 AD and 41 944 controls) and GWAX2018 (27 696 proxy maternal AD and 260 980 controls, 14 338 paternal AD and 245 941 controls) (GWAS+GWAX2022b). They identified 24 genome-wide significant loci [16], and all these loci were previously reported by GWAS+GWAX2018 [10], GWAS+GWAX2019 [11] or GWAS+GWAX2021b [14].

Collectively, AD GWAX and GWAS+GWAX have been widely reported [9–16]. All these AD GWAX datasets are based on UK Biobank participants [9, 10, 12], and all AD GWAS+GWAX datasets include the AD GWAS dataset from IGAP and the GWAX dataset from UK Biobank [9–16]. Therefore, the GWAS and GWAS+GWAX datasets are dependent and have extensive sample overlap. Table 1 provides the demographic descriptions of these AD GWAS, GWAX and GWAS+GWAX datasets.

**Table 1:** Demographic descriptions of AD GWAS, GWAX and GWAS+GWAX datasets

Dataset	Source	Case	Control	All	Loci	Heritability (SE)	Diagnostic criteria	Tissue	Meta method
GWAS2013 [4]	IGAP 2013 stage 1 + 2	25 580	48 466	74 046	19	0.09 (0.02)	Diagnostic criteria	Tissue	Fixed-effects IVW
GWAS2019 [8]	IGAP 2019 stage 1 + 2	35 274	59 163	94 437	26	0.07 (0.01)	DSM-III-R, DSM-IV, NINCDS-ADRDA	Blood	Fixed-effects IVW
GWAX2017 [9]	UK Biobank	14 482 proxy AD	100 082	114 564	1	NA	NINCDS-ADRDA, DSM-III-R, DSM-IV,DSM-V	Blood	NA
GWAX2018 [10]	UK Biobank	42 034 proxy AD	272 244	314 278	6	NA	ICD	Blood	NA
GWAX2021 [12]	UK Biobank	53 042 proxy AD	355 900	408 942	13	NA	ICD	Blood	NA
GWAS+GWAX2017 [9]	IGAP 2013 stage 1 + 2, UK Biobank	40 062 including 25 580 AD and 14 482 proxy AD	148 548	188 610	25	NA	ICD	Blood	Fixed-effects IVW
GWAS+GWAX2018 [10]	IGAP 2013 stage 1 + 2, UK Biobank	67 614 including 25 580 AD and 42 034 proxy AD	320 710	388 324	26	0.03 (0.004)	DSM-III-R, DSM-IV, NINCDS-ADRDA, ICD	Blood	Fixed-effects IVW
GWAS+GWAX2019 [11]	IGAP 2013 stage 1, UK Biobank, PGC-ALZ, ADSP	71 880 including 24 087 AD and 47 793 proxy AD	383 378	455 258	29	0.06 (0.01) without UK Biobank 0.02 (0.003) with UK Biobank	DSM-III-R, DSM-IV, NINCDS-ADRDA, ICD	Blood	mvGWAMA
GWAS+GWAX2021a [12]	IGAP 2019 stage 1 and UK Biobank	75 024 including 21 982 AD and 53 042 proxy AD	397 844	472 868	33	NA	NINCDS-ADRDA, DSM-III-R, DSM-IV, DSM-V, ICD, NIA–AA	Blood	Fixed-effects IVW
GWAS+GWAX2021b [14]	IGAP 2019 stage 1, UK Biobank, DemGene, TwinGene, STSA, deCODE, Finngen, GR@ACE, HUNT, BioVU, Gothenburg, ANMerge	90 338 including 43 725 AD and 46 613 proxy AD	1 036 225	1 126 563	38	0.031 (0.0062) without UK Biobank	NINCDS-ADRDA,DSM-III-R,DSM-IV,DSM-V,ICD	Blood	mvGWAMA
GWAS+GWAX2021c [13]	IGAP 2019 stage 1 + 2, UK Biobank, GR@ACE, EADB, PGC-ALZ, AD and GBCS, NxC, ADDN	97 796 including 55 762 AD and 42 034 proxy AD	604 504	702 300	35	0.03 (0.004)	NINCDS-ADRDA, DSM-III-R, DSM-IV, DSM-V, ICD	Blood	Fixed-effects IVW
GWAS+GWAX2022a [15]	EADB, UK Biobank, ADGC, CHARGE, FinnGen	111 326 including 62 051 AD and 49 275 proxy AD	677 663	788 989	75	0.03 (0.003)	NINCDS-ADRDA, DSM-III-R, DSM-IV, DSM-V, ICD, NIA–AA	Blood	Fixed-effects IVW
GWAS+GWAX2022b [16]	IGAP 2019 stage 1, UK Biobank	64 016 including 21 982 AD and 42 034 proxy AD	314 188	378 204	25	0.0695 (0.03)	NINCDS-ADRDA, DSM-III-R, DSM-IV, DSM-V, ICD, NIA–AA	Blood	multivariate Genomic SEM model

GWAS, genome-wide association studies; GWAX, GWAS by proxy; GWAS+GWAX, meta-analysis of GWAS and GWAX; IGAP, International Genomics of Alzheimer’s Project; IVW, inverse variance-weighted; mvGWAMA, multivariate GWAS meta-analysis; Heritability, SNP-based heritability on liability scale (5% prevalence) from John Hardy and colleagues [17] excluding GWAS+GWAX2022b; NA, not available; DSM, Diagnostic and Statistical Manual of Mental Disorders; ICD, International Classification of Diseases; NINCDS-ADRDA, National Institute of Neurological and Communicative Disorders and Stroke-Alzheimer Disease and Related Disorders Association; NIA-AA, National Institute on Aging and Alzheimer’s Association.

### AD risk loci across GWAS and GWAS+GWAX

A total of 82 AD susceptibility loci achieved genome-wide significance in at least one AD GWAS, including GWAS2019, and eight GWAS+GWAX datasets, including GWAS+GWAX2017, GWAS+GWAX2018, GWAS+GWAX2019, GWAS+GWAX2021a, GWAS+GWAX2021b, GWAS+GWAX2021c, GWAS+GWAX2022a and GWAS+GWAX2022b, as provided in Table 2. 32 (39%) of the 82 AD susceptibility loci were genome-wide significant only in a single dataset, without replication at the genome-wide significance level in other datasets (Table 2). Therefore, 50 (61%) loci were replicated in at least 2 of these 9 studies, and only 14 (17%) loci were replicated across all these 9 AD GWAS and GWAS+GWAX datasets, including *PICALM*, *EPHA1*, *CR1*, *CASS4*, *SORL1*, *CD2AP*, *ZCWPW1*, *ABCA7*, *ECHDC3*, *MS4A6A*, *SLC24A4*, *CLU*, *INPP5D* and *BIN1*, as provided in Table 2. This ratio of 17% is much lower than the earlier reported 37.5% that 15 out of the 40 AD susceptibility loci reached genome-wide significance across the AD GWAS, GWAX and GWAS+GWAX datasets [18].

**Table 2:** 82 AD loci identified by AD GWAS and GWAS+GWAX datasets

Dataset	SNP	Chr	Position	EA	NEA	OR	95%CI	*P*-value	Gene
Locus 1: 1p36.33									
GWAS+GWAX2021b	rs113020870	1	985377	T	C	NA	NA	3.83E-08	*AGRN*
Locus 2: 1p13.3									
GWAS+GWAX2022a	rs141749679	1	109888432	C	T	1.38	1.24–1.54	7.54E-09	*SORT1*
Locus 3: 1q23.3									
GWAS+GWAX2019	rs4575098	1	161155392	A	G	1.016	1.011–1.021	2.05E-10	*ADAMTS4*
GWAS+GWAX2021a	rs4575098	1	161155392	A	G	1.06	1.04–1.09	4.30E-08	*ADAMTS4*
Locus 4: 1q32.2									
GWAS+GWAX2017	rs6656401	1	207692049	A	G	1.17	NA	1.16E-26	*CR1*
GWAS+GWAX2018	rs6656401	1	207692049	A	G	1.15	1.12–1.18	1.37E-29	*CR1*
GWAS+GWAX2021a	rs679515	1	207750568	T	C	1.13	1.1–1.16	1.40E-23	*CR1*
GWAS+GWAX2021b	rs679515	1	207750568	C	T	NA	NA	2.42E-25	*CR1*
GWAS+GWAX2022a	rs679515	1	207750568	T	C	1.13	1.11–1.15	7.16E-46	*CR1*
GWAS+GWAX2019	rs2093760	1	207786828	A	G	1.024	1.019–1.029	1.10E-18	*CR1*
GWAS2019	rs4844610	1	207802552	A	C	1.17	1.13–1.21	3.60E-24	*CR1*
GWAS+GWAX2021c	rs4844610	1	207802552	A	C	1.14	1.11–1.16	7.30E-31	*CR1*
GWAS+GWAX2022b	rs679515	1	207750568	T	C	1.08	1.06–1.10	2.23E-23	*CR1*
Locus 5: 2p25.1									
GWAS+GWAX2022a	rs72777026	2	9699011	G	A	1.06	1.04–1.08	2.72E-08	*ADAM17*
Locus 6: 2p22.2									
GWAS+GWAX2021c	rs2192939	2	37484726	A	G	1.05	1.03–1.07	2.67E-08	*PRKD3*
GWAS+GWAX2022a	rs17020490	2	37531939	C	T	1.06	1.04–1.08	3.29E-09	*PRKD3*
Locus 7: 2p14									
GWAS+GWAX2021a	rs268134	2	65608363	A	G	1.06	1.04–1.09	1.54E-08	*SPRED2*
Locus 8: 2q12.2									
GWAS+GWAX2021b	rs115186657	2	106235428	C	A	NA	NA	1.33E-08	*NCK2*
GWAS+GWAX2021a	rs143080277	2	106366056	T	C	0.59	0.51–0.69	1.28E-12	*NCK2*
GWAS+GWAX2022a	rs143080277	2	106366056	C	T	1.47	1.33–1.63	2.07E-13	*NCK2*
Locus 9: 2q14.3									
GWAS+GWAX2017	rs6733839	2	127892810	T	C	1.19		3.09E-46	*BIN1*
GWAS+GWAX2019	rs4663105	2	127891427	C	A	1.031	1.026–1.035	3.38E-44	*BIN1*
GWAS+GWAX2021b	rs4663105	2	127891427	C	A	NA	NA	3.92E-58	*BIN1*
GWAS+GWAX2018	rs6733839	2	127892810	T	C	1.2	1.17–1.22	2.37E-69	*BIN1*
GWAS2019	rs6733839	2	127892810	T	C	1.2	1.17–1.23	2.10E-44	*BIN1*
GWAS+GWAX2021a	rs6733839	2	127892810	T	C	1.17	1.15–1.19	1.10E-54	*BIN1*
GWAS+GWAX2021c	rs6733839	2	127892810	C	T	0.84	0.83–0.86	6.43E-77	*BIN1*
GWAS+GWAX2022a	rs6733839	2	127892810	T	C	1.17	1.16–1.19	6.06E-118	*BIN1*
GWAS+GWAX2022b	rs6733839	2	127892810	C	T	0.91	0.90–0.92	4.82E-53	*BIN1*
Locus 10: 2q21.3									
GWAS+GWAX2021a	rs35564151	2	135372951	A	G	0.95	0.93–0.97	5.24E-08	*TMEM163*
Locus 11: 2q33.2									
GWAS+GWAX2022a	rs139643391	2	202878716	T	TC	0.94	0.92–0.96	1.08E-08	*WDR12*
Locus 12: 2q37.1									
GWAS2019	rs10933431	2	233981912	G	C	0.91	0.88–0.94	3.40E-09	*INPP5D*
GWAS+GWAX2019	rs10933431	2	233981912	G	C	0.985	0.98–0.99	8.92E-10	*INPP5D*
GWAS+GWAX2021a	rs10933431	2	233981912	C	G	1.08	1.05–1.11	1.41E-10	*INPP5D*
GWAS+GWAX2021c	rs10933431	2	233981912	C	G	1.08	1.06–1.10	3.64E-12	*INPP5D*
GWAS+GWAX2022a	rs10933431	2	233981912	G	C	0.93	0.92–0.95	3.62E-18	*INPP5D*
GWAS+GWAX2018	rs35349669	2	234068476	T	C	1.07	1.05–1.09	3.58E-11	*INPP5D*
GWAS+GWAX2017	rs35349669	2	234068476	C	T	0.93	NA	8.23E-09	*INPP5D*
GWAS+GWAX2021b	rs7597763	2	234082577	C	A	NA	NA	4.65E-09	*INPPD5*
GWAS+GWAX2022b	rs10933431	2	233981912	G	C	0.95	0.94–0.97	8.84E-10	*INPPD5*
Locus 13: 3p14.3									
GWAS+GWAX2019	rs184384746	3	57226150	T	C	1.215	1.136–1.299	1.24E-08	*HESX1*
Locus 14: 3q25.2									
GWAS+GWAX2022a	rs16824536	3	154787511	A	G	0.92	0.89–0.95	3.63E-08	*MME*
GWAS+GWAX2022a	rs61762319	3	154801978	G	A	1.16	1.11–1.21	2.16E-11	*MME*
Locus 15: 4p16.3									
GWAS+GWAX2022a	rs3822030	4	987343	G	T	0.95	0.94–0.96	8.29E-12	*IDUA*
Locus 16: 4p16.1									
GWAS+GWAX2021b	rs4504245	4	11014822	A	G	NA	NA	5.23E-12	*CLNK*
GWAS+GWAX2022a	rs6846529	4	11025131	C	T	1.07	1.05–1.08	2.20E-17	*CLNK*
GWAS+GWAX2019	rs6448453	4	11026028	A	G	1.014	1.01–1.019	1.93E-09	*CLNK*
GWAS+GWAX2021a	rs4351014	4	11027619	T	C	1.07	1.05–1.1	2.59E-11	*CLNK*
GWAS+GWAX2021c	rs4351014	4	11027619	C	T	0.94	0.92–0.96	5.37E-10	*HS3ST1*
GWAS+GWAX2022b	rs4351014	4	11027619	T	C	1.04	1.03–1.06	5.71E-10	*CLNK*
Locus 17: 4p14									
GWAS+GWAX2022a	rs2245466	4	40198846	G	C	1.05	1.03–1.06	1.22E-09	*RHOH*
Locus 18: 5p15.2									
GWAS+GWAX2022a	rs112403360	5	14724413	A	T	1.09	1.06–1.12	2.27E-09	*ANKH*
Locus 19: 5q14.3									
GWAS+GWAX2017	rs190982	5	88223420	G	A	0.93	NA	2.48E-08	*MEF2C*
GWAS+GWAX2022a	rs62374257	5	86223195	C	T	1.07	1.05–1.09	1.38E-15	*COX7C*
Locus 20: 5q31.3									
GWAS+GWAX2017	rs2074612	5	139714690	T	C	1.08	NA	8.00E-09	*HBEGF*
Locus 21: 5q33.1									
GWAS+GWAX2021b	rs871269	5	150432388	T	C	NA	NA	1.37E-09	*TNIP1*
GWAS+GWAX2022a	rs871269	5	150432388	T	C	0.96	0.95–0.97	8.67E-09	*TNIP1*
Locus 22: 5q33.3									
GWAS+GWAX2021b	rs6891966	5	156526331	A	G	NA	NA	7.91E-10	*HAVCR2*
Locus 23: 5q35.3									
GWAS+GWAX2022a	rs113706587	5	179628150	A	G	1.09	1.07–1.12	2.22E-16	*RASGEF1C*
Locus 24: 6p21.32									
GWAS+GWAX2017	rs9271087	6	32576170	C	A	1.10	NA	1.72E-11	*MTCO3P1*
GWAS+GWAX2017	rs9272561	6	32607141	G	A	1.11	NA	1.65E-08	*MTCO3P1*
GWAS+GWAX2021a	rs36096565	6	32560025	A	G	1.11	1.08–1.14	2.88E-15	*HLA-DRB1*
GWAS2019	rs9271058	6	32575406	A	T	1.1	1.07–1.13	1.40E-11	*HLA-DRB1*
GWAS+GWAX2022a	rs6605556	6	32583099	G	A	0.91	0.9–0.93	7.07E-20	*HLA-DQA1*
GWAS+GWAX2019	rs6931277	6	32583357	T	A	0.981	0.976–0.987	8.41E-11	*HLA-DRB1*
GWAS+GWAX2021b	rs1846190	6	32583813	A	G	NA	NA	2.66E-14	*HLA-DRB1*
GWAS+GWAX2021c	rs9275152	6	32652196	C	T	0.88	0.85–0.91	4.17E-16	*HLA*
Locus 25: 6p21.1									
GWAS+GWAX2019	rs187370608	6	40942196	A	G	1.254	1.189–1.323	1.45E-16	*TREM2*
GWAS+GWAX2021a	rs187370608	6	40942196	A	G	2.22	1.9–2.59	1.83E-23	*TREM2*
GWAS+GWAX2021b	rs187370608	6	40942196	A	G	NA	NA	1.26E-25	*TREM2*
GWAS+GWAX2022a	rs10947943	6	41004093	A	G	0.94	0.93–0.96	1.13E-09	*UNC5CL*
GWAS2019	rs114812713	6	41034000	C	G	1.32	1.22–1.42	2.10E-13	*OARD1*
GWAS+GWAX2022a	rs143332484	6	41129207	T	C	1.41	1.32–1.5	2.78E-25	*TREM2*
GWAS2019	rs75932628	6	41129252	T	C	2.08	1.73–2.49	2.70E-15	*TREM2*
GWAS+GWAX2021c	rs75932628	6	41129252	C	T	0.5	0.41–0.60	6.95E-14	*TREM2*
GWAS+GWAX2022a	rs75932628	6	41129252	T	C	2.39	2.09–2.73	2.53E-37	*TREM2*
GWAS+GWAX2022a	rs60755019	6	41149008	G	A	1.55	1.33–1.8	2.07E-08	*TREML2*
GWAS+GWAX2018	rs9381040	6	41154650	T	C	0.94	0.92–0.96	1.55E-08	*TREML2*
GWAS+GWAX2021c	rs9381040	6	41154650	C	T	1.05	1.03–1.07	3.72E-08	*TREML2*
GWAS+GWAX2022b	rs114812713	6	41034000	G	C	0.88	0.85–0.91	4.88E-12	*TREML2*
Locus 26: 6p12.3									
GWAS+GWAX2017	rs10948363	6	47487762	G	A	1.10	NA	2.75E-12	*CD2AP*
GWAS2019	rs9473117	6	47431284	C	A	1.09	1.06–1.12	1.20E-10	*CD2AP*
GWAS+GWAX2018	rs9381563	6	47432637	T	C	0.93	0.91–0.95	5.83E-14	*CD2AP*
GWAS+GWAX2019	rs9381563	6	47432637	C	T	1.014	1.01–1.019	2.52E-10	*CD2AP*
GWAS+GWAX2021c	rs9381564	6	47443806	A	G	0.93	0.91–0.94	1.79E-15	*CD2AP*
GWAS+GWAX2022a	rs7767350	6	47485126	T	C	1.08	1.06–1.09	7.94E-22	*CD2AP*
GWAS+GWAX2021b	rs9369716	6	47552180	T	A	NA	NA	1.70E-17	*CD2AP*
GWAS+GWAX2021a	rs1385742	6	47595155	A	T	1.07	1.05–1.09	1.11E-11	*CD2AP*
GWAS+GWAX2022b	rs1385742	6	47595155	A	T	1.05	1.03–1.06	9.12E-12	*CD2AP*
Locus 27: 6q21-q22.1									
GWAS+GWAX2022a	rs785129	6	114612895	T	C	1.04	1.03–1.06	2.40E-09	*HS3ST5*
Locus 28: 7p21.3									
GWAS+GWAX2022a	rs6943429	7	7856894	T	C	1.05	1.03–1.06	1.03E-10	*UMAD1*
GWAS+GWAX2022a	rs10952097	7	8244012	T	C	1.07	1.05–1.1	6.81E-09	*ICA1*
GWAS+GWAX2021b	rs5011436	7	12268758	C	A	NA	NA	2.70E-09	*TMEM106B*
GWAS+GWAX2022a	rs13237518	7	12269593	A	C	0.96	0.94–0.97	4.88E-11	*TMEM106B*
Locus 29: 7p15.2-p15.1									
GWAS+GWAX2022a	rs1160871	7	28129126	G	GTCTT	0.95	0.93–0.97	9.83E-09	*JAZF1*
Locus 30: 7p14.1									
GWAS+GWAX2017	rs2718058	7	37841534	G	A	0.94		1.90E-08	*EPDR1*
GWAS+GWAX2022a	rs6966331	7	37883793	T	C	0.96	0.94–0.97	4.64E-10	*EPDR1*
Locus 31: 7p12.2									
GWAS+GWAX2021a	rs2168589	7	50270105	A	T	1.06	1.03–1.08	7.68E-08	*IKZF1*
Locus 32: 7p11.2									
GWAS+GWAX2022a	rs76928645	7	54941328	T	C	0.93	0.91–0.95	1.62E-10	*SEC61G*
Locus 33: 7q22.1									
GWAS+GWAX2017	rs34995835	7	99990364	T	G	0.92		2.28E-11	*PILRA*
GWAS+GWAX2021b	rs7384878	7	99932049	C	T	NA	NA	9.41E-16	*ZCWPW1/ NYAP1*
GWAS+GWAX2022a	rs7384878	7	99932049	C	T	0.92	0.91–0.94	1.06E-26	*ZCWPW1/ NYAP1*
GWAS+GWAX2019	rs1859788	7	99971834	A	G	0.982	0.978–0.987	2.22E-15	*ZCWPW1*
GWAS+GWAX2021a	rs1859788	7	99971834	A	G	0.91	0.9–0.93	3.28E-18	*PILRA*
GWAS+GWAX2021c	rs1859788	7	99971834	A	G	0.92	0.90–0.93	1.94E-20	*PILRA*
GWAS+GWAX2018	rs1476679	7	100004446	T	C	1.1	1.07–1.12	9.93E-19	*ZCWPW1*
GWAS2019	rs12539172	7	100091795	T	C	0.92	0.90–0.95	9.30E-10	*NYAP1*
GWAS+GWAX2022b	rs60738304	7	100012579	A	C	0.95	0.94–0.97	3.07E-13	*ZCWPW1*
Locus 34: 7q34-q35									
GWAS+GWAX2017	rs11771145	7	143110762	A	G	0.92	NA	2.24E-12	*EPHA1*
GWAS+GWAX2018	rs10808026	7	143099133	A	C	0.91	0.89–0.93	1.13E-14	*EPHA1*
GWAS2019	rs10808026	7	143099133	A	C	0.9	0.88–0.93	1.30E-10	*EPHA1*
GWAS+GWAX2021c	rs56402156	7	143103481	A	G	0.92	0.90–0.94	9.63E-14	*EPHA1*
GWAS+GWAX2021b	rs3935067	7	143104331	C	G	NA	NA	4.69E-11	*EPHA1*
GWAS+GWAX2021a	rs12703526	7	143107588	T	G	1.07	1.05–1.09	9.63E-12	*EPHA1*
GWAS+GWAX2019	rs7810606	7	143108158	T	C	0.986	0.982–0.99	3.59E-11	*EPHA1*
GWAS+GWAX2022a	rs11771145	7	143110762	A	G	0.95	0.93–0.96	3.30E-14	*EPHA1*
GWAS+GWAX2022b	rs56402156	7	143103481	G	A	1.05	1.03–1.07	1.30E-10	*EPHA1*
Locus 35: 7q35									
GWAS+GWAX2019	rs114360492	7	145950029	T	C	1.19	1.124–1.259	2.10E-09	*CNTNAP2*
Locus 36: 8p23.1									
GWAS+GWAX2022a	rs1065712	8	11702122	C	G	1.09	1.06–1.12	1.94E-09	*CTSB*
Locus 37: 8p21.2									
GWAS+GWAX2017	rs28834970	8	27195121	C	T	1.10		4.26E-16	*PTK2B*
GWAS2019	rs73223431	8	27219987	T	C	1.1	1.07–1.13	6.30E-14	*PTK2B*
GWAS+GWAX2021c	rs73223431	8	27219987	C	T	0.93	0.91–0.94	1.94E-17	*PTK2B*
GWAS+GWAX2022a	rs73223431	8	27219987	T	C	1.07	1.06–1.08	4.03E-22	*PTK2B*
Locus 38: 8p21.1									
GWAS+GWAX2017	rs9331896	8	27467686	C	T	0.88		1.95E-25	*CLU*
GWAS+GWAX2018	rs4236673	8	27464929	A	G	0.9	0.88–0.91	1.07E-28	*CLU*
GWAS+GWAX2019	rs4236673	8	27464929	A	G	0.981	0.976–0.985	2.61E-19	*CLU*
GWAS+GWAX2022a	rs11787077	8	27465312	T	C	0.91	0.9–0.92	1.70E-44	*CLU*
GWAS+GWAX2021b	rs1532278	8	27466315	C	T	NA	NA	1.57E-22	*CLU*
GWAS2019	rs9331896	8	27467686	C	T	0.88	0.85–0.90	4.60E-24	*CLU*
GWAS+GWAX2021c	rs9331896	8	27467686	C	T	0.9	0.89–0.92	3.77E-29	*CLU*
GWAS+GWAX2021a	rs867230	8	27468503	A	C	1.11	1.09–1.13	7.71E-26	*CLU*
GWAS+GWAX2022b	rs867230	8	27468503	C	A	0.94	0.93–0.95	6.13E-22	*CLU*
Locus 39: 8q24.3									
GWAS+GWAX2021b	rs61732533	8	145108151	A	G	NA	NA	3.14E-09	*SHARPIN*
GWAS+GWAX2021c	rs34173062	8	145158607	A	G	1.16	1.11–1.21	1.33E-11	*SHARPIN*
GWAS+GWAX2022a	rs34173062	8	145158607	A	G	1.13	1.09–1.16	1.72E-16	*SHARPIN*
Locus 40: 9q31.1									
GWAS+GWAX2022a	rs1800978	9	107665978	G	C	1.06	1.04–1.08	1.59E-09	*ABCA1*
Locus 41: 10p14									
GWAS+GWAX2019	rs11257238	10	11717397	C	T	1.013	1.008–1.017	1.26E-08	*ECHDC3*
GWAS+GWAX2021b	rs7912495	10	11718713	G	A	NA	NA	7.68E-15	*USP6NL/ ECHDC3*
GWAS+GWAX2022a	rs7912495	10	11718713	G	A	1.06	1.05–1.08	9.74E-19	*USP6NL*
GWAS+GWAX2018	rs7920721	10	11720308	A	G	0.94	0.92–0.95	3.17E-11	*ECHDC3*
GWAS2019	rs7920721	10	11720308	G	A	1.08	1.06–1.11	1.80E-11	*ECHDC3*
GWAS+GWAX2021a	rs7920721	10	11720308	A	G	0.94	0.92–0.95	1.08E-11	*ECHDC3*
GWAS+GWAX2021c	rs7920721	10	11720308	A	G	0.94	0.93–0.96	3.09E-11	*ECHDC3*
GWAS+GWAX2017	rs7920721	10	11720308	G	A	1.07	NA	4.27E-08	*ECHDC3*
GWAS+GWAX2022b	rs7920721	10	11720308	A	G	0.96	0.95–0.97	3.98E-11	*ECHDC3*
Locus 42: 10q21.2									
GWAS+GWAX2021a	rs1171814	10	61645833	T	G	0.95	0.93–0.97	3.80E-08	*CCDC6*
GWAS+GWAX2021b	rs7902657	10	61738152	T	G	NA	NA	3.68E-08	*CCDC6*
GWAS+GWAX2022a	rs7068231	10	61784928	T	G	0.95	0.94–0.96	3.32E-13	*ANK3*
Locus 43: 10q23.1									
GWAS+GWAX2022a	rs6586028	10	82253984	C	T	0.93	0.91–0.94	1.97E-19	*TSPAN14*
GWAS+GWAX2021a	rs1878036	10	82280137	T	G	0.93	0.91–0.95	2.74E-09	*TSPAN14*
Locus 44: 10q24.1									
GWAS+GWAX2022a	rs6584063	10	98026407	G	A	0.89	0.86–0.92	6.73E-11	*BLNK*
Locus 45: 10q26.13									
GWAS+GWAX2022a	rs7908662	10	124172912	G	A	0.96	0.95–0.97	2.59E-09	*PLEKHA1*
Locus 46:11p11.2									
GWAS+GWAX2017	rs12292911	11	47449072	A	G	1.08	NA	2.08E-11	*PSMC3*
GWAS2019	rs3740688	11	47380340	G	T	0.92	0.89–0.94	5.40E-13	*SPI1*
GWAS+GWAX2021b	rs3740688	11	47380340	T	G	NA	NA	8.78E-09	*MADD/SPI1*
GWAS+GWAX2021c	rs3740688	11	47380340	T	G	1.07	1.05–1.09	1.93E-14	*SPI1*
GWAS+GWAX2021a	rs10437655	11	47391948	A	G	1.07	1.05–1.09	6.91E-11	*SPI1*
GWAS+GWAX2022a	rs10437655	11	47391948	A	G	1.06	1.04–1.07	5.28E-14	*SPI1*
GWAS+GWAX2018	rs12292911	11	47449072	A	G	1.06	1.04–1.08	3.30E-09	*SPI1*
GWAS+GWAX2022b	rs3740688	11	47380340	G	T	0.96	0.95–0.97	6.77E-11	*SPI1*
Locus 47:11q12.1									
GWAS+GWAX2017	rs983392	11	59923508	G	A	0.90		3.57E-17	*MS4A6A*
GWAS2019	rs7933202	11	59936926	C	A	0.89	0.87–0.92	1.90E-19	*MS4A2*
GWAS+GWAX2019	rs2081545	11	59958380	A	C	0.983	0.979–0.987	1.55E-15	*MS4A6A*
GWAS+GWAX2018	rs1582763	11	60021948	A	G	0.92	0.9–0.93	1.01E-18	*MS4A4A*
GWAS+GWAX2021b	rs1582763	11	60021948	A	G	NA	NA	3.40E-33	*MS4A4A*
GWAS+GWAX2021c	rs1582763	11	60021948	A	G	0.91	0.90–0.93	8.69E-24	*MS4A4A*
GWAS+GWAX2022a	rs1582763	11	60021948	A	G	0.91	0.9–0.92	3.74E-42	*MS4A4A*
GWAS+GWAX2021a	rs72924626	11	60095740	T	C	1.09	1.07–1.11	9.33E-20	*MS4A4A*
GWAS+GWAX2022b	rs1582763	11	60021948	G	A	1.06	1.04–1.07	4.46E-19	*MS4A4A*
Locus 48:11q14.2									
GWAS+GWAX2017	rs10792832	11	85867875	A	G	0.88		1.58E-25	*FNTAL1*
GWAS+GWAX2019	rs867611	11	85776544	G	A	0.98	0.976–0.985	2.19E-18	*PICALM*
GWAS+GWAX2021b	rs561655	11	85800279	A	G	NA	NA	1.24E-26	*PICALM*
GWAS+GWAX2018	rs10792832	11	85867875	A	G	0.88	0.87–0.9	5.08E-36	*PICALM*
GWAS+GWAX2021a	rs10792832	11	85867875	A	G	0.9	0.88–0.92	5.21E-26	*PICALM*
GWAS2019	rs3851179	11	85868640	T	C	0.88	0.86–0.90	6.00E-25	*PICALM*
GWAS+GWAX2021c	rs3851179	11	85868640	C	T	1.12	1.10–1.14	3.87E-38	*PICALM*
GWAS+GWAX2022a	rs3851179	11	85868640	T	C	0.9	0.89–0.92	2.95E-48	*PICALM*
GWAS+GWAX2022b	rs3851179	11	85868640	T	C	0.94	0.93–0.95	6.64E-26	*PICALM*
Locus 49:11q24.1									
GWAS+GWAX2022a	rs74685827	11	121353077	G	T	1.19	1.13–1.25	2.81E-11	*SORL1*
GWAS+GWAX2018	rs11218343	11	121435587	T	C	1.24	1.18–1.3	4.58E-17	*SORL1*
GWAS2019	rs11218343	11	121435587	C	T	0.8	0.75–0.85	2.90E-12	*SORL1*
GWAS+GWAX2019	rs11218343	11	121435587	C	T	0.966	0.956–0.976	1.09E-11	*SORL1*
GWAS+GWAX2021a	rs11218343	11	121435587	T	C	1.2	1.15–1.26	5.59E-14	*SORL1*
GWAS+GWAX2021b	rs11218343	11	121435587	C	T	NA	NA	1.33E-13	*SORL1*
GWAS+GWAX2021c	rs11218343	11	121435587	C	T	0.83	0.79–0.87	5.59E-16	*SORL1*
GWAS+GWAX2022a	rs11218343	11	121435587	C	T	0.84	0.81–0.87	1.40E-21	*SORL1*
GWAS+GWAX2017	rs11218343	11	121435587	C	T	0.78		3.98E-16	*SORL1*
GWAS+GWAX2022b	rs11218343	11	121435587	T	C	1.11	1.08–1.15	6.95E-12	*SORL1*
Locus 50:12q24.13									
GWAS+GWAX2022a	rs6489896	12	113719788	C	T	1.08	1.05–1.1	1.80E-09	*TPCN1*
Locus 51:14q22.1									
GWAS+GWAX2021b	rs7146179	14	53298853	A	G	NA	NA	6.99E-11	*FERMT2*
GWAS+GWAX2018	rs17125924	14	53391680	A	G	0.9	0.87–0.92	1.34E-11	*FERMT2*
GWAS2019	rs17125924	14	53391680	G	A	1.14	1.09–1.18	1.40E-09	*FERMT2*
GWAS+GWAX2021a	rs17125924	14	53391680	A	G	0.9	0.87–0.93	3.69E-10	*FERMT2*
GWAS+GWAX2021c	rs17125924	14	53391680	A	G	0.89	0.87–0.92	6.30E-14	*FERMT2*
GWAS+GWAX2022a	rs17125924	14	53391680	G	A	1.1	1.07–1.12	8.32E-16	*FERMT2*
GWAS+GWAX2022b	rs17125924	14	53391680	A	G	0.94	0.92–0.96	1.06E-09	*FERMT2*
Locus 52:14q32.12									
GWAS+GWAX2017	rs10498633	14	92926952	T	G	0.92	NA	4.20E-09	*SLC24A4*
GWAS+GWAX2022a	rs7401792	14	92931261	G	A	1.04	1.02–1.05	4.83E-08	*SLC24A4*
GWAS2019	rs12881735	14	92932828	C	T	0.92	0.89–0.94	7.40E-09	*SLC24A4*
GWAS+GWAX2021c	rs11623019	14	92936971	C	T	0.93	0.91–0.94	2.41E-13	*SLC24A4*
GWAS+GWAX2018	rs12590654	14	92938855	A	G	0.92	0.9–0.95	8.21E-12	*SLC24A4*
GWAS+GWAX2019	rs12590654	14	92938855	A	G	0.986	0.981–0.99	1.65E-10	*SLC24A4*
GWAS+GWAX2021a	rs12590654	14	92938855	A	G	0.93	0.91–0.95	7.45E-14	*SLC24A4*
GWAS+GWAX2021b	rs12590654	14	92938855	A	G	NA	NA	6.63E-17	*SLC24A4*
GWAS+GWAX2022a	rs12590654	14	92938855	A	G	0.93	0.92–0.95	4.25E-21	*SLC24A4*
GWAS+GWAX2022b	rs12590654	14	92938855	G	A	1.05	1.03–1.06	7.10E-13	*SLC24A4*
Locus 53:14q32.33									
GWAS+GWAX2022a	rs7157106	14	106228095	A	G	1.05	1.03–1.07	1.99E-08	*IGHG3*
GWAS+GWAX2022a	rs10131280	14	107121607	A	G	0.94	0.92–0.96	4.26E-10	*IGHV3–64*
Locus 54:15q21.2									
GWAS+GWAX2021a	rs12592778	15	50992311	A	G	0.93	0.91–0.95	1.74E-09	*SPPL2A*
GWAS+GWAX2022a	rs8025980	15	50994011	G	A	0.96	0.94–0.97	1.32E-08	*SPPL2A*
GWAS+GWAX2018	rs59685680	15	51001534	T	G	1.07	1.05–1.1	9.17E-09	*SPPL2A*
GWAS+GWAX2017	rs59685680	15	51001534	G	T	0.92		7.32E-09	*SPPL2A*
Locus 55:15q21.3									
GWAS+GWAX2019	rs442495	15	59022615	C	T	0.987	0.982–0.991	1.31E-09	*ADAM10*
GWAS+GWAX2021a	rs442495	15	59022615	T	C	1.07	1.05–1.09	2.67E-11	*ADAM10*
GWAS+GWAX2018	rs593742	15	59045774	A	G	1.07	1.05–1.09	2.78E-11	*ADAM10*
GWAS2019	rs593742	15	59045774	G	A	0.93	0.91–0.95	6.80E-09	*ADAM10*
GWAS+GWAX2021c	rs593742	15	59045774	A	G	1.08	1.06–1.10	3.20E-15	*ADAM10*
GWAS+GWAX2021b	rs602602	15	59057023	A	T	NA	NA	6.22E-15	*ADAM10*
GWAS+GWAX2022a	rs602602	15	59057023	A	T	0.94	0.93–0.96	2.07E-15	*ADAM10*
GWAS+GWAX2022b	rs442495	15	59022615	T	C	1.04	1.03–1.06	9.19E-11	*ADAM10*
Locus 56:15q22.2									
GWAS+GWAX2019	rs117618017	15	63569902	T	C	1.018	1.011–1.024	3.35E-08	*APH1B*
GWAS+GWAX2021a	rs117618017	15	63569902	T	C	1.09	1.06–1.12	1.05E-08	*APH1B*
GWAS+GWAX2021b	rs117618017	15	63569902	T	C	NA	NA	7.00E-12	*APH1B*
GWAS+GWAX2021c	rs117618017	15	63569902	C	T	0.91	0.89–0.94	7.98E-11	*APH1B*
GWAS+GWAX2022a	rs117618017	15	63569902	T	C	1.11	1.09–1.13	2.15E-25	*APH1B*
GWAS+GWAX2022b	rs117618017	15	63569902	C	T	0.95	0.93–0.97	2.02E-08	*APH1B*
Locus 57:15q22.31									
GWAS+GWAX2022a	rs3848143	15	64423506	G	A	1.05	1.04–1.07	8.41E-11	*SNX1*
Locus 58:15q25.1									
GWAS+GWAX2022a	rs12592898	15	79229199	A	G	0.94	0.92–0.96	4.18E-09	*CTSH*
Locus 59:16p12.3									
GWAS2019	rs7185636	16	19808163	C	T	0.92	0.89–0.95	2.40E-08	*IQCK*
GWAS+GWAX2021c	rs7185636	16	19808163	C	T	0.95	0.93–0.97	2.30E-05	*IQCK*
Locus 60:16p11.2									
GWAS+GWAX2022a	rs1140239	16	30021402	T	C	0.94	0.93–0.96	2.59E-13	*DOC2A*
GWAS+GWAX2018	rs889555	16	31122571	T	C	0.94	0.92–0.96	4.11E-08	*KAT8*
GWAS+GWAX2022a	rs889555	16	31122571	T	C	0.95	0.94–0.97	1.96E-11	*KAT8*
GWAS+GWAX2021a	rs2884738	16	31126321	A	C	0.94	0.92–0.96	4.47E-09	*KAT8*
GWAS+GWAX2019	rs59735493	16	31133100	A	G	0.987	0.983–0.992	3.98E-08	*KAT8*
Locus 61:16q22.1									
GWAS+GWAX2018	rs4985556	16	70694000	A	C	1.09	1.05–1.12	3.67E-08	*IL34*
GWAS+GWAX2021c	rs4985556	16	70694000	A	C	1.08	1.05–1.11	2.28E-08	*IL34*
GWAS+GWAX2022a	rs4985556	16	70694000	A	C	1.07	1.05–1.09	5.98E-10	*IL34*
Locus 62:16q23.2									
GWAS2019	rs62039712	16	79355857	A	G	1.16	1.10–1.23	3.70E-08	*WWOX*
GWAS+GWAX2022a	rs450674	16	79608408	C	T	0.96	0.95–0.98	3.16E-08	*MAF*
Locus 63:16q23.3									
GWAS+GWAX2022a	rs12446759	16	81773003	G	A	0.95	0.94–0.96	1.22E-13	*PLCG2*
GWAS+GWAX2018	rs12444183	16	81773209	A	G	0.95	0.93–0.97	3.15E-08	*PLCG2*
GWAS+GWAX2021a	rs12444183	16	81773209	A	G	0.95	0.93–0.97	5.46E-08	*PLCG2*
GWAS+GWAX2021c	rs12444183	16	81773209	A	G	0.95	0.93–0.97	1.48E-08	*PLCG2*
GWAS+GWAX2022a	rs72824905	16	81942028	G	C	0.74	0.68–0.81	8.48E-12	*PLCG2*
Locus 64:16q24.1									
GWAS+GWAX2022a	rs16941239	16	86454210	A	T	1.13	1.08–1.17	1.29E-08	*FOXF1*
Locus 65:16q24.3									
GWAS+GWAX2022a	rs56407236	16	90170095	A	G	1.11	1.08–1.14	6.47E-15	*PRDM7*
Locus 66:17p13.3									
GWAS+GWAX2022a	rs35048651	17	1728046	T	TGAG	1.06	1.04–1.08	7.67E-11	*WDR81*
Locus 67:17p13.2									
GWAS+GWAX2021c	rs72835017	17	4746294	A	G	0.91	0.89–0.94	1.36E-09	*MINK1/ CHRNE*
GWAS+GWAX2021b	rs7209200	17	4969940	T	C	NA	NA	3.18E-08	*SCIMP/ RABEP1*
GWAS+GWAX2021a	rs61182333	17	5133128	T	C	1.09	1.06–1.12	1.35E-09	*SCIMP*
GWAS+GWAX2018	rs7225151	17	5137047	A	G	1.1	1.07–1.13	6.06E-12	*SCIMP*
GWAS+GWAX2022a	rs7225151	17	5137047	A	G	1.08	1.05–1.1	4.13E-13	*SCIMP*
GWAS+GWAX2021c	rs75511804	17	5138304	C	T	0.91	0.89–0.93	5.16E-13	*SCIMP*
GWAS+GWAX2019	rs113260531	17	5138980	A	G	1.02	1.013–1.026	9.16E-10	*SCIMP*
GWAS+GWAX2017	rs77493189	17	5118951	G	T	1.11		9.60E-10	*SCIMP*
GWAS+GWAX2022b	rs75511804	17	5138304	C	T	0.95	0.93–0.97	7.47E-09	*SCIMP*
Locus 68:17p11.2									
GWAS+GWAX2022a	rs2242595	17	18059454	A	G	0.94	0.92–0.96	1.11E-09	*MYO15A*
Locus 69:17q21.31-q21.32									
GWAS+GWAX2022a	rs5848	17	42430244	T	C	1.07	1.06–1.09	2.38E-20	*GRN*
GWAS+GWAX2021b	rs708382	17	42442344	C	T	NA	NA	1.98E-09	*GRN*
GWAS+GWAX2021c	rs386572859	17	44368216	A	G	0.95	0.91–0.98	1.07E-03	*MAPT/KANSL1*
GWAS+GWAX2022a	rs199515	17	44856641	G	C	0.94	0.93–0.96	9.34E-13	*WNT3*
GWAS+GWAX2022a	rs616338	17	47297297	T	C	1.32	1.23–1.42	2.82E-14	*ABI3*
GWAS+GWAX2019	rs28394864	17	47450775	A	G	1.012	1.008–1.016	1.87E-08	*ABI3*
GWAS+GWAX2021b	rs28394864	17	47450775	A	G	NA	NA	4.90E-10	*ABI3*
Locus 70:17q22									
GWAS+GWAX2021a	rs2526378	17	56404349	A	G	1.05	1.03–1.07	3.07E-07	*TSPOAP1*
GWAS+GWAX2021b	rs2632516	17	56409089	C	G	NA	NA	7.46E-10	*TSPOAP1-AS1*
GWAS+GWAX2022a	rs2526377	17	56410041	G	A	0.95	0.94–0.97	1.58E-12	*TSPOAP1*
Locus 71:17q23.3									
GWAS+GWAX2018	rs138190086	17	61538148	A	G	1.25	1.16–1.35	1.95E-09	*ACE*
GWAS2019	rs138190086	17	61538148	A	G	1.3	1.19–1.42	5.30E-09	*ACE*
GWAS+GWAX2021b	rs6504163	17	61545779	C	T	NA	NA	1.23E-09	*ACE*
GWAS+GWAX2022a	rs4277405	17	61548918	C	T	0.94	0.93–0.95	8.80E-20	*ACE*
GWAS+GWAX2021a	rs4311	17	61560763	T	C	0.95	0.93–0.97	1.21E-08	*ACE*
GWAS+GWAX2021c	rs4311	17	61560763	C	T	1.06	1.04–1.08	5.51E-10	*ACE*
GWAS+GWAX2022b	rs138190086	17	61538148	G	A	0.88	0.84–0.92	2.03E-08	*ACE*
Locus 72:18q21.31									
GWAS+GWAX2019	rs76726049	18	56189459	C	T	1.057	1.036–1.078	3.30E-08	*ALPK2*
Locus 73:19p13.3									
GWAS+GWAX2017	rs3752231	19	1043638	T	C	1.11	NA	8.85E-12	*ABCA7*
GWAS+GWAX2017	rs4147929	1	1063443	A	G	1.14	NA	4.35E-17	*ABCA7*
GWAS+GWAX2019	rs111278892	19	1039323	G	C	1.02	1.014–1.025	7.93E-11	*ABCA7*
GWAS+GWAX2018	rs3752231	19	1043638	T	C	1.09	1.07–1.12	4.37E-13	*ABCA7*
GWAS+GWAX2021c	rs3752231	19	1043638	C	T	0.92	0.90–0.94	8.42E-16	*ABCA7*
GWAS+GWAX2021a	rs12151021	19	1050874	A	G	1.08	1.06–1.1	2.41E-13	*ABCA7*
GWAS+GWAX2021b	rs12151021	19	1050874	A	G	NA	NA	2.81E-15	*ABCA7*
GWAS+GWAX2022a	rs12151021	19	1050874	A	G	1.1	1.09–1.12	1.59E-37	*ABCA7*
GWAS2019	rs3752246	19	1056492	G	C	1.15	1.11–1.18	3.10E-16	*ABCA7*
GWAS+GWAX2022b	rs3795065	19	1039444	C	T	1.05	1.03–1.06	1.74E-11	*ABCA7*
Locus 74:19p13.3									
GWAS+GWAX2022a	rs149080927	19	1854254	G	GC	1.05	1.04–1.07	5.09E-10	*KLF16*
Locus 75:19q13.32									
GWAS+GWAX2018	rs41289512	19	45351516	C	G	0.4	0.38–0.42	6.70E-255	*APOE*
GWAS+GWAX2019	rs41289512	19	45351516	G	C	1.222	1.208–1.235	5.79E-276	*APOE*
GWAS2019	rs429358	19	45411941	C	T	3.32	3.20–3.45	1.20E-881	*APOE*
GWAS+GWAX2021a	rs429358	19	45411941	T	C	0.31	0.3–0.32	0	*APOE*
GWAS+GWAX2021b	rs429358	19	45411941	C	T	NA	NA	<1.0e-300	*APOE*
GWAS+GWAX2021c	rs429358	19	45411941	C	T	3	2.93–3.07	0	*APOE4*
GWAS+GWAX2021c	rs7412	19	45412079	C	T	1.58	1.52–1.65	3.20E-123	*APOE2*
GWAS+GWAX2019	rs76320948	19	46241841	T	C	1.034	1.022–1.047	4.64E-08	*AC074212.3*
GWAS+GWAX2022b	rs429358	19	45411941	T	C	0.53	0.52–0.53	0	*APOE*
Locus 76:19q13.33									
GWAS+GWAX2021b	rs2452170	19	49213504	A	G	NA	NA	1.72E-08	*NTN5*
GWAS+GWAX2022a	rs9304690	19	50453317	T	C	1.05	1.03–1.07	4.74E-09	*SIGLEC11*
Locus 77:19q13.41									
GWAS+GWAX2019	rs3865444	19	51727962	A	C	0.987	0.982–0.991	6.34E-09	*CD33*
GWAS+GWAX2021a	rs3865444	19	51727962	A	C	0.94	0.92–0.96	1.29E-08	*CD33*
GWAS+GWAX2018	rs12459419	19	51728477	T	C	0.94	0.92–0.96	7.97E-09	*CD33*
GWAS+GWAX2021c	rs12459419	19	51728477	C	T	1.06	1.04–1.08	3.11E-08	*CD33*
GWAS+GWAX2021b	rs1354106	19	51737991	G	T	NA	NA	2.21E-10	*CD33*
GWAS+GWAX2017	rs12459419	19	51728477	T	C	0.92	NA	8.58E-09	*CD33*
GWAS+GWAX2022b	rs3865444	19	51727962	C	A	1.04	1.03–1.05	9.18E-09	*CD33*
Locus 78:19q13.42									
GWAS+GWAX2022a	rs587709	19	54771451	C	T	1.05	1.04–1.07	3.63E-11	*LILRB2*
GWAS+GWAX2021b	rs1761461	19	54825174	C	A	NA	NA	1.56E-09	*LILRB2*
Locus 79:20p13									
GWAS+GWAX2022a	rs1358782	20	393978	A	G	0.95	0.94–0.97	1.55E-08	*RBCK1*
Locus 80:20q13.31									
GWAS+GWAX2017	rs7274581	20	55018260	C	T	0.89	NA	1.22E-08	*CASS4*
GWAS+GWAX2018	rs6069736	20	54983075	T	C	0.89	0.86–0.93	2.00E-10	*CASS4*
GWAS+GWAX2021b	rs6069737	20	54995699	T	C	NA	NA	6.73E-16	*CASS4*
GWAS2019	rs6024870	20	54997568	A	G	0.88	0.85–0.92	3.50E-08	*CASS4*
GWAS+GWAX2021c	rs6024870	20	54997568	A	G	0.9	0.87–0.93	2.16E-11	*CASS4*
GWAS+GWAX2019	rs6014724	20	54998544	G	A	0.978	0.971–0.985	6.56E-10	*CASS4*
GWAS+GWAX2021a	rs6014724	20	54998544	A	G	1.12	1.08–1.15	1.07E-10	*CASS4*
GWAS+GWAX2022a	rs6014724	20	54998544	G	A	0.89	0.87–0.91	4.13E-21	*CASS4*
GWAS+GWAX2022b	rs6014724	20	54998544	A	G	1.07	1.05–1.09	5.75E-10	*CASS4*
Locus 81:20q13.33									
GWAS+GWAX2022a	rs6742	20	62374441	T	C	0.95	0.93–0.97	2.58E-09	*SLC2A4RG*
Locus 82:21q21.3									
GWAS+GWAX2021c	rs2154481	21	27473875	C	T	0.95	0.93–0.96	9.26E-10	*APP*
GWAS+GWAX2022a	rs2154481	21	27473875	C	T	0.95	0.94–0.97	1.00E-12	*APP*
GWAS+GWAX2021b	rs2154482	21	27520931	T	G	NA	NA	7.66E-10	*APP*
GWAS+GWAX2021a	rs2830489	21	28148191	T	C	0.94	0.92–0.96	3.09E-08	*ADAMTS1*
GWAS+GWAX2022a	rs2830489	21	28148191	T	C	0.95	0.94–0.97	1.69E-10	*ADAMTS1*
GWAS2019	rs2830500	21	28156856	A	C	0.93	0.91–0.96	2.60E-08	*ADAMTS1*

Chr, Chromosome; AD, Alzheimer’s disease; NA, not available; GWAS, genome-wide association studies; GWAX, GWAS for family history of disease, known as GWAS by proxy; GWAS+GWAX, meta-analysis of GWAS and GWAX; The position is based on GRCh37/hg19.

Theoretically, GWAS+GWAX could improve the statistical power and facilitate the identification of more significant genetic variants by increasing the large-scale sample size [9–15]. However, it seems that AD GWAS+GWAX findings do not support the hypothesis, as provided in Tables 1 and 2. GWAS+GWAX2018 had predominant proxy AD (42 034 versus 14 482) and controls (320 710 versus 148 548), but identified a similar number of genome-wide significant genetic variants with GWAS+GWAX2017 (26 versus 25), as provided in Table 2. GWAS+GWAX2021b has the largest sample size *n* = 1 126 563 (90 338 cases, including 43 725 clinically diagnosed AD, 46 613 proxy AD and 1 036 225 controls) among the seven GWAS+GWAX datasets and only identified 38 genome-wide significant AD susceptibility loci, as provided in Table 2. Compared with GWAS+GWAX2021b, GWAS+GWAX2022a only included 788 989 individuals (111 326 cases, including 62 051 clinically diagnosed AD and 49 275 proxy AD and 677 663 controls), but identified 75 genome-wide significant AD susceptibility loci (Table 2). Importantly, GWAS+GWAX2022a, but not GWAS+GWAX2021, identified the most significant signals for the majority of the 82 loci and especially for 13 of these 14 loci (excluding *SORL1*) that were repeated among all the 9 AD GWAS and GWAS+GWAX datasets (Table 2).

A possible explanation is that GWAS+GWAX2021b achieved the largest sample size primarily by increasing the number of population-based controls, and GWAS+GWAX2022a achieved a larger effective sample size by predominantly increasing the number of clinically diagnosed AD, which may have contributed to enhanced statistical power to identify more genetic variants [20]. In fact, GWAS+GWAX2021c also consists of a larger number of clinically diagnosed AD than GWAS+GWAX2021b and has similar numbers of clinically diagnosed AD, proxy AD and controls as GWAS+GWAX2022a, as provided in Table 2. However, only 35 loci were identified by GWAS+GWAX2021c. These findings suggest that the effective sample size may not explain this phenomenon.

## METHODS

### Meta-analysis methods in AD GWAS+GWAX

Until now, three meta-analysis methods have been used in AD GWAS+GWAX, including fixed effects inverse variance-weighted, mvGWAMA (multivariate GWAS meta-analysis) and multivariate genomic SEM model, as provided in Table 1. Fixed effects inverse variance-weighted is a widely used meta-analysis method in both AD GWAS, including GWAS2013 [4] and GWAS2019 [8], and AD GWAS+GWAX, including GWAS+GWAX2017 [9], GWAS+GWAX2018 [10], GWAS+GWAX2021a [12], GWAS+GWAX2021c [13] and GWAS+GWAX2022a [15]. We assume that $k$ represents the number of studies, ${x}_i$ represents the effect estimate (e.g. a log odds ratio), ${s}_i$ is the standard error, ${\left({s}_i\right)}^2$ is the variance of ${x}_i$, ${w}_i$ is the weight of study $i$ ($i=1,\cdots, k$), and ${w}_i=\frac{1}{{\left({s}_i\right)}^2}$ [21]. Under the fixed effects models, the inverse variance-weighted effect size estimate can be calculated as 


(1) \begin{equation*}\overline{X}=\sum_{i=1}^k{w}_i{x}_i\Big/\sum_{i=1}^k{w}_i. \end{equation*}

The variance and standard error of $\overline{X}$ are $\operatorname{var}\left(\overline{X}\right)=\frac{1}{\sum_{i=1}^k{w}_i}$ and $\mathrm{SE}\left(\overline{X}\right)=\frac{1}{\sqrt{\sum_{i=1}^k{w}_i}}$, respectively. $\overline{X}$ follows a normal distribution, and the *Z*-score can be calculated as 


(2) \begin{equation*}Z=\frac{\overline{X}}{SE\left(\overline{X}\right)}. \end{equation*}

mvGWAMA, a meta-analysis method that incorporates sample size weighting of *Z* statistics, which has been used in GWAS+GWAX2019 [11] and GWAS+GWAX2021b [14]. mvGWAMA is designed specifically to perform a GWAS meta-analysis when there is sample overlap using a sample size-weighted method [11, 14]. The UK Biobank data was generated with a continuous phenotype that was the number of parents with AD (*n* = 0, 1, 2) in both GWAS+GWAX2019 [11] and GWAS+GWAX2021b [14]. UK Biobank individuals with phenotype values = 0 are taken as controls, and ≥1 are taken as proxy AD cases [11, 14]. For the analysis of genome-wide association, logistic regression was conducted to compare dichotomous phenotypes (specifically, clinically diagnosed AD cases versus controls), while linear regression was employed to analyze continuous phenotypes (proxy AD cases versus proxy controls for the UK Biobank cohort) [11, 14]. Therefore, the effect estimate from the UK Biobank is based on a continuous phenotype and is not comparable to the effect estimate from other datasets [11, 14]. Jansen *et al.* developed the sample size-weighted method mvGWAMA to meta-analyze UK Biobank GWAX and other GWAS datasets [11, 14].

Both fixed effects inverse variance-weighted meta-analysis and sample size weighted meta-analysis often produce dramatically downwardly biased common variant SNP heritability of AD [16]. GWAS+GWAX2022b introduces a novel approach named the multivariate genomic SEM model to combine the AD GWAS and GWAX datasets [16]. In contrast to inverse variance weighted meta-analysis or sample size-weighted meta-analysis, the multivariate genomic SEM model relaxes standard assumptions and yields unbiased estimates, leading to a significant increase in the heritability of AD associated with common variant SNPs [16]. Meanwhile, the multivariate genomic SEM model provides little evidence of genome-wide heterogeneity across AD GWAS, maternal GWAX and paternal GWAX using a SNP-specific heterogeneity statistic (*Q*_SNP_) after adjusting for the empirically derived attenuation coefficients (λ) [16].

### Heterogeneity test methods

Until now, both Cochran’s *Q* statistic and ${I}^2$ statistic have been widely utilized to assess statistical heterogeneity across multiple GWAS datasets for complex human traits, especially AD [4, 8, 15]. Cochran’s *Q* statistic and ${I}^2$ statistic have different properties [22]. Cochran’s *Q* statistic consistently increases with both the number of studies and the size of the studies [22]. ${I}^2$ statistic always increases with the size of studies, but not the number of studies [22]. Cochran’s *Q* statistic approximately follows a chi-square distribution with *k*−1 degrees of freedom (*k* represents the number of studies) [22, 23]. *Q* is given by:


(3) \begin{equation*} Q=\sum_{i=1}^k{w}_i{\left({x}_i-\sum_{i=1}^k{w}_i{x}_i\Big/\sum_{i=1}^k{w}_i\right)}^2. \end{equation*}

${I}^2$ is derived from Cochran’s *Q* statistic, which is calculated by:


(4) \begin{equation*} {I}^2=\max \left\{0,\frac{Q-\left(k-1\right)}{Q}\right\}. \end{equation*}

${I}^2$ varies from 0 to 100%, with intervals of 0–25%, 25–50%, 50–75% and 75–100% representing low, moderate, large and extreme heterogeneity, respectively [21, 22].

## STATISTICAL HETEROGENEITY IN AD GENETIC FINDINGS

### AD GWAS findings in the context of statistical heterogeneity

IGAP2013 represents a large-scale meta-analysis encompassing 4 GWAS cohorts in stage 1, which includes 17 008 individuals with AD and 37 154 controls, and 11 GWAS cohorts in stage 2, with 8572 AD cases and 11 312 controls [4]. The heterogeneity test conducted using Cochran’s *Q* statistic and ${I}^2$ statistic shows that 18 genetic variants exhibit no evidence of heterogeneity across these 15 GWAS cohorts with ${I}^2$ < 25% and Cochran’s *Q* test *P* > 0.05, as detailed in Supplementary Table SS1 [4]. Only the *BIN1* rs6733839 variant showed moderate heterogeneity with ${I}^2$ = 28% and Cochran’s *Q* test *P* = 0.061 in IGAP2013 stages 1 and 2 [4].

IGAP2019 is a comprehensive large-scale meta-analysis encompassing 4 GWAS datasets in stage 1, 10 GWAS datasets in stage 2 and 4 GWAS datasets in stage 3. The heterogeneity test employing both Cochran’s *Q* statistic and ${I}^2$ statistic indicates that 24 genetic variants (excluding *APOE* rs429358 variant as this region was not carried forward to the replication stage) show no evidence of heterogeneity across these 18 GWAS cohorts with ${I}^2$ < 25% and Cochran’s *Q* test *P* > 0.05, as provided in Supplementary Table SS2. Only the *MS4A2* rs7933202 variant had moderate heterogeneity with ${I}^2$ = 27% and Cochran’s *Q* test *P* = 0.05 in IGAP2019 stages 1 and 2 [8]. It is worth mentioning that three genetic variants, rs7185636 (*P* = 1.10E-01), rs62039712 (*P* = 1.30E-01) and rs2830500 (*P* = 1.30E-01), which showed no significant association with AD (*P* > 0.05) in IGAP2019 Stage 3A or Stage 3B, still have no evidence of statistical heterogeneity, as detailed in Supplementary Table SS2 [8].

### AD GWAS+GWAX findings in context of statistical heterogeneity

We select both Cochran’s *Q* statistic and ${I}^2$ statistic to assess the statistical heterogeneity of GWAS+GWAX findings in five GWAS+GWAX datasets, including GWAS+GWAX2017, GWAS+ GWAX2018, GWAS+GWAX2021a, GWAS+GWAX2021c and GWAS+ GWAX2022a, as fixed effects inverse variance-weighted meta-analysis is employed in these studies. All these GWAS+GWAX findings showed evidence of statistical heterogeneity, especially for GWAS+GWAX2018, as provided in Table 3 and Supplementary Tables S3–S7.

**Table 3:** Heterogeneity test of AD GWAS and GWAS+GWAX findings

Dataset	SNP	Heterogeneity test *I^2^*(%)				Chi-square test	
		[0–25]	(25–50]	(50–75]	(75–100]	OR (95%CI)	*P*-value
GWAS2013 [4]	19	18	1	0	0	–	–
GWAS2019 [8]	25	24	1	0	0	0.75 (0.04–12.82)	1
GWAS+GWAX2017 [9]	25	13	5	2	5	16.62 (1.91–144.24)	6.07E-03
GWAS+GWAX2018 [10]	26	1	2	2	21	450 (26.36–7681.35)	6.97E-09
GWAS+GWAX2021a [12]	33	16	3	8	6	19.13 (2.28–160.34)	2.12E-03
GWAS+GWAX2021c [13]	36	21	2	9	4	12.86 (1.54–107.11)	1.19E-02
GWAS+GWAX2022a [15]	83	56	19	8	0	8.68 (1.1–68.46)	3.42E-02

AD, Alzheimer’s disease; NA, not available; GWAS, genome-wide association studies; GWAX, GWAS for family history of disease, known as GWAS by proxy; GWAS+GWAX, meta-analysis of GWAS and GWAX; NA, not available. AD, Alzheimer’s disease; OR, odds ratio; CI, confidence Interval; OR is based on the number of genetic variants with ${I}^2>25\%$  *versus* the number of genetic variants with ${I}^2\le 25\%$.

A total of 96% (25), 88% (23) and 81% (21) of these 26 genetic variants exhibited evidence of statistical heterogeneity with ${I}^2$ > 25%, ${I}^2$ > 50% and ${I}^2$ > 75% in GWAS+GWAX2018 (Table 3). 48% (12), 28% (7) and 20% (5) of the 25 genetic variants demonstrated evidence of statistical heterogeneity with ${I}^2$ > 25%, ${I}^2$ > 50% and ${I}^2$ > 75% in GWAS+GWAX2017 (Table 3). Importantly, GWAS+GWAX2022a had the lowest percent of genetic variants, showing evidence of statistical heterogeneity. 33% (27), 10% (8) and 0% (0) of the 83 genetic variants suggested evidence of statistical heterogeneity with ${I}^2$ > 25%, ${I}^2$ > 50% and ${I}^2$ > 75% in GWAS+GWAX2022a (Table 3). Compared with GWAS2013, the chi-square (χ2) test indicates that all these GWAS+GWAX findings but not GWAS2019 have significantly increased numbers of genetic variants showing the evidence of statistical heterogeneity (Table 3). Importantly, GWAS+GWAX2018 has the maximum odds ratio (OR) = 450 and GWAS+GWAX2022a has the minimum OR = 8.68 using the number of genetic variants with ${I}^2$ > 25% versus the number of genetic variants with ${I}^2$ ≤ 25%.

It is known that the fixed-effects model assumes consistency in the effects of risk alleles across datasets, making it the preferred method for synthesizing GWAS datasets [21]. However, the model can be biased when significant heterogeneity exists among the datasets [21]. Evidence shows that statistical heterogeneity across studies reduces statistical power and predictive accuracy in large-scale GWAS for multiple complex human traits, further diminishing the number of genome-wide significant loci and heritability [24]. Take years of education, for example, evidence has shown a significant reduction in the number of genome-wide significant loci, ranging from a relative loss of 51–62%, and a corresponding decrease in heritability, with an *R*^2^ loss of 36–38% [24]. Therefore, the widely present statistical heterogeneity across the AD GWAS+GWAX findings may attenuate the statistical power, contribute to the missing heritability, and may further explain that: (1) GWAS+GWAX2018 had predominant proxy AD (42 034 versus 14 482) and controls (320 710 versus 148 548) but only identified a similar number of genome-wide significant genetic variants as GWAS+GWAX2017 (26 versus 25); (2) GWAS+GWAX2022a identified the most significant signals for the majority of the 82 loci and the maximum number of genome-wide significant genetic variants; and (3) heritability decreased as the sample size increased, as provided in Table 1.

Theoretically, if increased statistical heterogeneity reduces the statistical power, then a decrease in statistical heterogeneity may increase the statistical power in GWAS+GWAX. Interestingly, genetic findings from GWAS+GWAX2022b support this hypothesis. Using multivariate genomic SEM model, GWAS+GWAX2022b highlighted little evidence of genome-wide heterogeneity across AD GWAS, maternal GWAX and paternal GWAX datasets after adjusting for the empirically derived attenuation coefficients (λ) [16]. Meanwhile, GWAS+GWAX2022b substantially increased the common variant SNP heritability of AD outside of the *APOE* region to 7–11% and identified 24 genome-wide significant loci [16]. Importantly, all these loci were reported by GWAS+GWAX2018, GWAS+GWAX2019 or GWAS+GWAX2021b [16]. These findings show that a decrease in statistical heterogeneity may contribute to identify genome-wide significant loci and increase the common variant SNP heritability of AD.

### Potential sources of statistical heterogeneity in AD GWAS+GWAX

First, the increased number of studies and the size of studies may have caused the statistical heterogeneity in AD GWAS+GWAX. It is known that all AD GWAS+GWAX datasets are based on a large-scale meta-analysis of multiple AD GWAS cohorts and UK Biobank cohort, as presented in Table 1. Here, we employed both Cochran’s *Q* statistic and ${I}^2$ statistic to rigorously assess statistical heterogeneity across multiple AD GWAS and GWAS+GWAX datasets. Cochran’s *Q* statistic always increases as both the number of studies and the size of studies increase [22]. ${I}^2$ statistic always increases as the size of studies increases, but not the number of studies [22]. ${\tau}^2$ or ${\mathrm{Tau}}^2$ serve as an estimator of the variance among studies and is derived from Cochran’s *Q* statistic [21, 22], which is defined by


(5) \begin{equation*} {\tau}^2=\max \left\{0,\frac{Q-\left(k-1\right)}{^{\sum_{i=1}^k{w}_i{-}^{\sum_{i=1}^k{w_i}^2}}\Big/ {}_{\sum_{i=1}^k{w}_i}}\right\}. \end{equation*}

${\tau}^2$ does not systematically increase with either the number of studies, or the size of studies [22]. Until now, there is still no exact criterion to define heterogeneity using ${\tau}^2$, as it is difficult to interpret [21, 22]. ${\tau}^2$ is widely used in two ways. On one hand, ${\tau}^2$ is used to compute its square root (i.e. $\tau$), which can be interpreted as an estimate of the standard deviation of underlying effects across studies [22]. On the other hand, ${\tau}^2$ is used to assign weights to the studies in the meta-analysis under the random-effects models [22]. Under the random-effects models, ${w_i}^R=\frac{1}{\frac{1}{w_i}+{\tau}^2}=\frac{1}{{\left({s}_i\right)}^2+{\tau}^2}$, the inverse-variance-weighted effect-size estimate can be calculated as $\overline{X^R}=\sum_{i=1}^k{w^R}_i{x}_i/\sum_{i=1}^k{w^R}_i$. The variance and standard error of $\overline{X^R}$ are $\operatorname{var}\left(\overline{X^R}\right)=\frac{1}{\sum_{i=1}^k{w^R}_i}$ and $\mathrm{SE}\left(\overline{X^R}\right)=\frac{1}{\sqrt{\sum_{i=1}^k{w^R}_i}}$. $\overline{X^R}$ follows a normal distribution, and the *Z*-score can be calculated as


(6) \begin{equation*} {Z}^R=\frac{\overline{X^R}}{\mathrm{SE}\left(\overline{X^R}\right)}. \end{equation*}

The original studies have offered more comprehensive information about fixed-effects models and random-effects models [21, 25].

Second, phenotypic heterogeneity between clinically diagnosed AD and proxy AD might have led to statistical heterogeneity in AD GWAS+GWAX, as proxy AD is often less precise than clinical diagnoses due to a lack of distinction between AD and other dementia subtypes [18]. In the UK Biobank, the status of AD and control participants was assessed via self-report [10]. Participants were asked to report, ‘Has/did your father or mother ever suffer from Alzheimer’s disease/dementia?’ [10]. However, not all UK Biobank participants may be able to accurately discriminate AD from other dementia sub-types because of the diverse clinical presentations and underlying genetic complexities [10, 18]. The self-reported parental AD status may not accurately represent the clinically diagnosed AD status [10, 18]. The incorrect classification of AD status may have caused the phenotypic heterogeneity and further caused the statistical heterogeneity in AD GWAS+GWAX [10, 18, 24].

Manchia *et al.* [26] have investigated the influence of phenotypic heterogeneity on the statistical power of GWAS using both simulated and real data. They discovered that phenotypic heterogeneity, measured by the proportion of ‘non-cases’ (i.e. controls) within the case group, significantly decreased the statistical power to detect the genetic association and greatly reduced the genetic effect estimates [26]. Meanwhile, it is estimated that heterogeneity of 50% increases the required sample size by approximately threefold [26]. These findings indicate that precise phenotype delineation may be more crucial than increased sample size to detect the true genetic associations [26].

## CONCLUSIONS AND FUTURE DIRECTIONS

Here, we summarized recent 10 years of AD genetic findings from large-scale GWAS, GWAX and GWAS+GWAX since 2013 in participants of European ancestry. We highlighted that AD GWAS+GWAX with the largest sample size could not identify the most significant signals, the maximum number of genome-wide significant genetic variants and maximum heritability. We identified the widely present statistical heterogeneity across the AD GWAS+GWAX datasets but not in the AD GWAS datasets. We consider that statistical heterogeneity may have attenuated the statistical power in AD GWAS+GWAX and may contribute to explain: (1) the low repeatability (17%) of genome-wide significant AD susceptibility loci across all these AD GWAS and GWAS+GWAX datasets and (2) the decreased heritability (40–2%) as the increased sample size in GWAS+GWAX datasets.

Given the facts above, much work is required in future studies. First, it is necessary to combine the AD GWAS datasets from IGAP and other AD GWAS datasets specific to individuals of European ancestry for a meta-analysis, which may increase the statistical power by incorporating clinically or pathologically diagnosed cases and controls, reducing the phenotypic heterogeneity and further reducing the statistical heterogeneity. Second, AD GWAX and GWAS+GWAX findings should be interpreted with the utmost caution and require further detailed examination. Our recent study underscores the significant difference and statistical heterogeneity between clinically diagnosed AD GWAS and the self-report proxy phenotype GWAX [7]. Using AD GWAS dataset from GWAS2019 [8], we identified that every SD increase in years of schooling (about 3.6 years) was a 29% decrease in the risk of developing AD (OR = 0.71, 95% CI: 0.60–0.84 and *P* = 1.02E-04) [7]. Using AD GWAX dataset from GWAX2021, we found that for every SD increase in years of schooling, there was a significant 84% increase in the risk of developing AD (OR = 1.84, 95% CI: 1.59–2.13 and *P* = 4.66E-16) [7]. Importantly, our findings were further verified by two recent MR studies [27, 28]. Third, other meta-analysis methods may be used in AD GWAS+GWAX, such as multivariate genomic SEM model [16] and random-effects inverse variance-weighted meta-analysis [21]. The random-effects model assumes that the individual datasets’ true risk allele effects fluctuate around the overall mean effect, and it is particularly suited for the case in which there is heterogeneity [21]. In fact, the random-effects model is more appropriate than the fixed-effects model for assessing the generalizability of the observed association and estimating the average effect size of the associated variant, along with its uncertainty across diverse populations [21].

In fact, there are significant differences in AD genetic variants across different populations [29–34]. For instance, there is substantial evidence that demonstrates significant disparities in certain AD genetic variants between East Asian and European populations [30]. AD genetic variants in the Chinese population did not exhibit a significant association with AD in European population [29]. Here, we consider that statistical heterogeneity may have attenuated the statistical power in AD GWAS+GWAX, and may contribute to explain the low repeatability (17%) and the decreased heritability (40–2%). Therefore, we only choose the AD GWAS and GWAS+GWAX datasets for participants of European ancestry, as the majority of AD GWAS and GWAS+GWAX datasets from other ethnic groups apart from European ancestry are not publicly accessible. Our findings should be further verified in other populations besides those of European ancestry when the GWAS or GWAS+GWAX datasets become publicly available.

**Key Points**

- Alzheimer’s disease (AD) genetic findings from large-scale genome-wide association studies (GWAS), GWAS by proxy (GWAX) and meta-analysis of GWAS and GWAX (GWAS+GWAX) have been called into question because of very low repeatability of AD susceptibility loci and the low heritability of AD.
- We summarize recent 10 years of AD genetic findings and provide a new interpretation of these findings from the perspective of statistical heterogeneity.
- We highlighted that AD GWAS+GWAX with the largest sample size could not identify the most significant signals, maximum number of genome-wide significant genetic variants or maximum heritability.
- We identified the widely present statistical heterogeneity in the various AD GWAS+GWAX datasets, but not in AD GWAS datasets.

## Supplementary Material

Supplementary_Tables_bbae140

## Data Availability

All pertinent data are included within this paper. The authors affirm that all data that underlying the findings are freely accessible via consortia websites without any restrictions, or alternatively, can be obtained from the consortia upon reasonable request. The IGAP dataset is publicly available at: http://web.pasteur-lille.fr/en/recherche/u744/igap/igap_download.php, https://www.niagads.org/datasets/ng00075; GWAX2018 is available at: https://datashare.ed.ac.uk/handle/10283/3364, 3_UKB_AD_parental_meta_summary_output_June2019; GWAX2021 is available at: https://www.ebi.ac.uk/gwas/search?query=GCST90012878.

## Author Biographies

**Shan Gao** is a doctor student in Beijing Institute of Brain Disorders, Laboratory of Brain Disorders, Ministry of Science and Technology, Collaborative Innovation Center for Brain Disorders, Capital Medical University, Beijing 100069, China.

**Tao Wang** is a doctor student in Chinese Institute for Brain Research, Beijing, China.

**Zhifa Han** is a postdoctor in China-Japanese Friendship Hospital, Beijing, China.

**Yang Hu** is an associate professor in School of Computer Science and Technology, Harbin Institute of Technology, Harbin, China.

**Ping Zhu** is a doctor student in Beijing Institute of Brain Disorders, Laboratory of Brain Disorders, Ministry of Science and Technology, Collaborative Innovation Center for Brain Disorders, Capital Medical University, Beijing 100069, China.

**Yanli Xue** is an assistant professor in School of Biomedical Engineering, Capital Medical University, Beijing, China.

**Chen Huang** is an assistant professor in Dr. Neher's Biophysics Laboratory for Innovative Drug Discovery, State Key Laboratory of Quality Research in Chinese Medicine, Macau University of Science and Technology, Taipa, Macao SAR, 999078, China.

**Yan Chen** is an associate professor in Department of Epidemiology and Biostatistics, School of Public Health, Wannan Medical College, No. 22, Wenchang Road, Wuhu 241002, Anhui, China.

**Guiyou Liu** is a professor in Beijing Institute of Brain Disorders, Laboratory of Brain Disorders, Ministry of Science and Technology, Collaborative Innovation Center for Brain Disorders, Capital Medical University, Beijing 100069, China.
