# Supplementary material for: Interpretation of 10 years of Alzheimer’s disease genetic findings in the perspective of statistical heterogeneity
Source: Brief Bioinform. 2024 May 6;25(3):bbae140. doi: 10.1093/bib/bbae140 (PMC11074593; doi:10.1093/bib/bbae140)
Supplement: Supplementary_Tables_bbae140 [file supplementary_tables_bbae140.doc]

**Supplementary Table 1. Summary results of 19 AD GWAS genetic variants identified in GWAS2013 [1]**

| **SNP** | **Chr** | **Position (GRCh37/hg19)** | **Gene** | **EA** | **NEA** | **EAF** | **IGAP2013 Stage 1 + Stage 2** | | | **IGAP2013 Stage 1** | | | **IGAP2013 Stage 2** | | | **Heterogeneity** | |
| --- | --- | --- | --- | --- | --- | --- | --- | --- | --- | --- | --- | --- | --- | --- | --- | --- | --- |
|  |  |  |  |  |  |  | **OR** | **95 CI** | ***P* value** | **OR** | **95 CI** | ***P* value** | **OR** | **95 CI** | ***P* value** | ***I^2^*** | ***P* value** |
| rs6656401 | 1 | 207692049 | *CR1* | A | G | 0.197 | 1.18 | 1.14-1.22 | 5.69E-24 | 1.17 | 1.12–1.22 | 7.70E-15 | 1.21 | 1.14–1.28 | 7.90E-11 | 0 | 0.78 |
| rs6733839 | 2 | 127892810 | *BIN1* | T | C | 0.409 | 1.22 | 1.18-1.25 | 6.94E-44 | 1.21 | 1.17–1.25 | 1.70E-26 | 1.24 | 1.18–1.29 | 3.40E-19 | 28% | 0.061 |
| rs35349669 | 2 | 234068476 | *INPP5D* | T | C | 0.488 | 1.08 | 1.05-1.11 | 3.17E-08 | 1.07 | 1.03–1.10 | 9.60E-05 | 1.10 | 1.05–1.15 | 5.70E-05 | 0 | 0.80 |
| rs190982 | 5 | 88223420 | *MEF2C* | G | A | 0.408 | 0.93 | 0.90–0.95 | 3.2 0E-08 | 0.92 | 0.89–0.95 | 2.50E-06 | 0.93 | 0.89–0.98 | 3.40E-03 | 0 | 0.64 |
| rs9271192 | 6 | 32578530 | *HLA-DRB5* | C | A | 0.27 | 1.11 | 1.08-1.15 | 2.90E-12 | 1.11 | 1.07–1.16 | 1.60E-08 | 1.12 | 1.06–1.18 | 4.20E-05 | 0 | 0.54 |
| rs10948363 | 6 | 47487762 | *CD2AP* | G | A | 0.266 | 1.10 | 1.07-1.13 | 5.20E-11 | 1.10 | 1.07–1.14 | 3.10E-08 | 1.09 | 1.04–1.15 | 4.10E-04 | 0 | 0.90 |
| rs2718058 | 7 | 37841534 | *NME8* | G | A | 0.373 | 0.93 | 0.90–0.95 | 4.80E-09 | 0.93 | 0.90–0.96 | 1.30E-05 | 0.91 | 0.87–0.95 | 6.30E-05 | 0 | 0.92 |
| rs1476679 | 7 | 100004446 | *ZCWPW1* | C | T | 0.287 | 0.91 | 0.89-0.94 | 5.58E-10 | 0.92 | 0.89–0.96 | 7.40E-06 | 0.89 | 0.85–0.94 | 9.70E-06 | 0 | 0.70 |
| rs11771145 | 7 | 143110762 | *EPHA1* | A | G | 0.338 | 0.90 | 0.88-0.93 | 1.12E-13 | 0.90 | 0.87–0.93 | 8.80E-10 | 0.90 | 0.86–0.95 | 2.80E-05 | 14% | 0.24 |
| rs28834970 | 8 | 27195121 | *PTK2B* | C | T | 0.366 | 1.10 | 1.08-1.13 | 7.37E-14 | 1.10 | 1.07–1.14 | 3.30E-09 | 1.11 | 1.06–1.17 | 4.30E-06 | 10% | 0.30 |
| rs9331896 | 8 | 27467686 | *CLU* | C | T | 0.387 | 0.86 | 0.84-0.89 | 2.77E-25 | 0.86 | 0.84–0.89 | 9.60E-17 | 0.86 | 0.82–0.90 | 4.50E-10 | 0 | 0.49 |
| rs10838725 | 11 | 47557871 | *CELF1* | C | T | 0.316 | 1.08 | 1.05-1.11 | 1.12E-08 | 1.08 | 1.04–1.11 | 6.70E-06 | 1.09 | 1.04–1.14 | 4.1E-04 | 0 | 0.76 |
| rs983392 | 11 | 59923508 | *MS4A6A* | G | A | 0.391 | 0.90 | 0.87-0.92 | 6.14E-16 | 0.90 | 0.87–0.93 | 2.80E-11 | 0.90 | 0.86–0.94 | 4.50E-06 | 1% | 0.45 |
| rs10792832 | 11 | 85867875 | *PICALM* | A | G | 0.358 | 0.87 | 0.85-0.89 | 9.32E-26 | 0.88 | 0.85–0.91 | 6.50E-16 | 0.85 | 0.81–0.89 | 1.10E-11 | 0 | 0.98 |
| rs11218343 | 11 | 121435587 | *SORL1* | C | T | 0.039 | 0.77 | 0.72-0.82 | 9.73E-15 | 0.76 | 0.70–0.83 | 5.00E-11 | 0.78 | 0.70–0.88 | 4.00E-05 | 0 | 0.83 |
| rs17125944 | 14 | 53400629 | *FERMT2* | C | T | 0.092 | 1.14 | 1.09-1.19 | 7.95E-09 | 1.13 | 1.07–1.19 | 1.00E-05 | 1.17 | 1.08–1.26 | 1.60E-04 | 10% | 0.30 |
| rs10498633 | 14 | 92926952 | *SLC24A4* | T | G | 0.217 | 0.91 | 0.88-0.94 | 5.54E-09 | 0.90 | 0.87–0.94 | 1.50E-07 | 0.93 | 0.88–0.98 | 7.80E-03 | 0 | 0.63 |
| rs4147929 | 19 | 1063443 | *ABCA7* | A | G | 0.190 | 1.15 | 1.11-1.19 | 1.06E-15 | 1.14 | 1.10–1.20 | 1.70E-09 | 1.17 | 1.10–1.24 | 9.90E-08 | 0 | 0.94 |
| rs7274581 | 20 | 55018260 | *CASS4* | C | T | 0.083 | 0.88 | 0.84-0.92 | 2.46E-08 | 0.87 | 0.82–0.92 | 1.60E-06 | 0.89 | 0.82–0.96 | 4.10E-03 | 0 | 0.99 |

EA, effect allele; NEA, non-effect allele; EAF, effect allele frequency average in the discovery sample; *I^2^*, heterogeneity estimate; *I^2^* in GWAS2013 is calculated by GWAS2013 Stage 1 vs. GWAS2013 Stage 2 across different studies. Heterogeneity *P* is from Cochran’s Q test; Chr., chromosome; CI, confidence interval; OR, odds ratio;

**Supplementary Table 2. Summary results of 26 AD GWAS genetic variants identified in GWAS2019 [2]**

| **SNP** | **Chr** | **Position**  **(GRCh37/hg19)** | **Gene** | **EA** | **NEA** | **EAF** | **Stage 1 discovery (*n* = 63,926)** | | | **Stage 2 (*n* = 18,845)** | | | **Overall (*n* = 82,771)** | | | **Heterogeneity** | |
| --- | --- | --- | --- | --- | --- | --- | --- | --- | --- | --- | --- | --- | --- | --- | --- | --- | --- |
|  |  |  |  |  |  |  | **OR** | **95 CI** | ***P* value** | **OR** | **95 CI** | ***P* value** | **OR** | **95 CI** | ***P* value** | ***I^2^*** | ***P* value** |
| rs4844610 | 1 | 207802552 | *CR1* | A | C | 0.187 | 1.16 | 1.12–1.20 | 8.20E-16 | 1.2 | 1.13–1.27 | 3.80E-10 | 1.17 | 1.13–1.21 | 3.60E-24 | 0 | 0.8 |
| rs6733839 | 2 | 127892810 | *BIN1* | T | C | 0.407 | 1.18 | 1.15–1.22 | 4.00E-28 | 1.23 | 1.18–1.29 | 2.00E-18 | 1.2 | 1.17–1.23 | 2.10E-44 | 15% | 0.2 |
| rs10933431 | 2 | 233981912 | *INPP5D* | G | C | 0.223 | 0.9 | 0.87–0.94 | 2.60E-07 | 0.92 | 0.87–0.97 | 3.20E-03 | 0.91 | 0.88–0.94 | 3.40E-09 | 0 | 0.8 |
| rs75932628 | 6 | 41129252 | *TREM2* | T | C | 0.008 | 2.01 | 1.65–2.44 | 2.90E-12 | 2.5 | 1.56–4.00 | 1.50E-04 | 2.08 | 1.73–2.49 | 2.70E-15 | 0 | 0.6 |
| rs9271058 | 6 | 32575406 | *HLA-DRB1* | A | T | 0.27 | 1.1 | 1.06–1.14 | 5.10E-08 | 1.11 | 1.06–1.17 | 5.70E-05 | 1.1 | 1.07–1.13 | 1.40E-11 | 10% | 0.3 |
| rs9473117 | 6 | 47431284 | *CD2AP* | C | A | 0.28 | 1.09 | 1.05–1.12 | 2.30E-07 | 1.11 | 1.05–1.16 | 1.00E-04 | 1.09 | 1.06–1.12 | 1.20E-10 | 0 | 0.6 |
| rs12539172 | 7 | 100091795 | *NYAP1* | T | C | 0.303 | 0.93 | 0.91–0.96 | 2.10E-05 | 0.89 | 0.84–0.93 | 2.10E-06 | 0.92 | 0.90–0.95 | 9.30E-10 | 0 | 0.8 |
| rs10808026 | 7 | 143099133 | *EPHA1* | A | C | 0.199 | 0.9 | 0.87–0.94 | 3.10E-08 | 0.91 | 0.86–0.96 | 1.10E-03 | 0.9 | 0.88–0.93 | 1.30E-10 | 0 | 0.5 |
| rs73223431 | 8 | 27219987 | *PTK2B* | T | C | 0.367 | 1.1 | 1.07–1.13 | 8.30E-10 | 1.11 | 1.06–1.16 | 1.50E-05 | 1.1 | 1.07–1.13 | 6.30E-14 | 0 | 0.6 |
| rs9331896 | 8 | 27467686 | *CLU* | C | T | 0.387 | 0.88 | 0.85–0.91 | 3.60E-16 | 0.87 | 0.83–0.91 | 1.70E-09 | 0.88 | 0.85–0.90 | 4.60E-24 | 3% | 0.4 |
| rs7920721 | 10 | 11720308 | *ECHDC3* | G | A | 0.389 | 1.08 | 1.05–1.11 | 1.90E-07 | 1.07 | 1.02–1.12 | 3.20E-03 | 1.08 | 1.05–1.11 | 2.30E-09 | 0 | 0.8 |
| rs3740688 | 11 | 47380340 | *SPI1* | G | T | 0.448 | 0.91 | 0.89–0.94 | 9.70E-11 | 0.93 | 0.88–0.97 | 1.20E-03 | 0.92 | 0.89–0.94 | 5.40E-13 | 4% | 0.4 |
| rs7933202 | 11 | 59936926 | *MS4A2* | C | A | 0.391 | 0.89 | 0.86–0.92 | 2.20E-15 | 0.9 | 0.86–0.95 | 1.60E-05 | 0.89 | 0.87–0.92 | 1.90E-19 | 27% | 0.05 |
| rs3851179 | 11 | 85868640 | *PICALM* | T | C | 0.356 | 0.89 | 0.86–0.91 | 5.80E-16 | 0.85 | 0.81–0.89 | 6.10E-11 | 0.88 | 0.86–0.90 | 6.00E-25 | 0 | 0.8 |
| rs11218343 | 11 | 121435587 | *SORL1* | C | T | 0.04 | 0.81 | 0.76–0.88 | 2.70E-08 | 0.77 | 0.68–0.87 | 1.80E-05 | 0.8 | 0.75–0.85 | 2.90E-12 | 7% | 0.3 |
| rs17125924 | 14 | 53391680 | *FERMT2* | G | A | 0.093 | 1.13 | 1.08–1.19 | 6.60E-07 | 1.15 | 1.06–1.25 | 5.00E-04 | 1.14 | 1.09–1.18 | 1.40E-09 | 8% | 0.3 |
| rs12881735 | 14 | 92932828 | *SLC24A4* | C | T | 0.221 | 0.92 | 0.88–0.95 | 4.90E-07 | 0.92 | 0.87–0.97 | 4.30E-03 | 0.92 | 0.89–0.94 | 7.40E-09 | 0 | 0.6 |
| rs138190086 | 17 | 61538148 | *ACE* | A | G | 0.02 | 1.29 | 1.15–1.44 | 7.50E-06 | 1.41 | 1.18–1.69 | 1.80E-04 | 1.32 | 1.20–1.45 | 7.50E-09 | 0 | 0.9 |
| rs3752246 | 19 | 1056492 | *ABCA7* | G | C | 0.182 | 1.13 | 1.09–1.18 | 6.60E-10 | 1.18 | 1.11–1.25 | 4.70E-08 | 1.15 | 1.11–1.18 | 3.10E-16 | 0 | 0.5 |
| rs429358 | 19 | 45411941 | *APOE* | C | T | 0.216 | 3.32 | 3.20–3.45 | 1.20E-881 |  |  |  |  |  |  |  |  |
| rs6024870 | 20 | 54997568 | *CASS4* | A | G | 0.088 | 0.88 | 0.84–0.93 | 1.10E-06 | 0.9 | 0.82–0.97 | 9.00E-03 | 0.88 | 0.85–0.92 | 3.50E-08 | 0 | 0.9 |
| Stage 3A |  |  |  |  |  |  | Stage 1 + 2 (**n** = 82,771) | | | Stage 3A (**n** = 11,666) | | | Overall (**n** = 94,437) | | | ***I^2^*** | ***P* value** |
| rs593742 | 15 | 59045774 | *ADAM10* | G | A | 0.295 | 0.93 | 0.91–0.96 | 1.30E-07 | 0.91 | 0.85–0.98 | 1.50E-02 | 0.93 | 0.91–0.95 | 6.80E-09 | 12.3% | 0.31 |
| rs7185636 | 16 | 19808163 | *IQCK* | C | T | 0.18 | 0.92 | 0.89–0.95 | 8.40E-08 | 0.94 | 0.86–1.01 | 1.10E-01 | 0.92 | 0.89–0.95 | 2.40E-08 | 0 | 0.98 |
| **Stage 3B** |  |  |  |  |  |  | **Stage 1 (*n* = 63,926)** | | | **Stage 3B (*n* = 30,511)** | | | **Overall (*n* = 94,437)** | | | ***I^2^*** | ***P* value** |
| rs114812713 | 6 | 41034000 | *OARD1* | C | G | 0.03 | 1.35 | 1.24–1.47 | 4.50E-12 | 1.23 | 1.06–1.42 | 7.20E-03 | 1.32 | 1.22–1.42 | 2.10E-13 | 7.4% | 0.37 |
| rs62039712 | 16 | 79355857 | *WWOX* | A | G | 0.116 | 1.17 | 1.10–1.23 | 1.20E-07 | 1.14 | 0.96–1.36 | 1.30E-01 | 1.16 | 1.10–1.23 | 3.70E-08 | 0 | 0.87 |
| rs2830500 | 21 | 28156856 | *ADAMTS1* | A | C | 0.308 | 0.93 | 0.91–0.96 | 7.40E-08 | 0.95 | 0.89–1.02 | 1.30E-01 | 0.93 | 0.91–0.96 | 2.60E-08 | 0 | 0.91 |

EA, effect allele; NEA, non-effect allele; EAF, effect allele frequency average in the discovery sample; *I^2^*, heterogeneity estimate; *I^2^* in GWAS2019 is calculated by GWAS2019 Stage 1 vs. GWAS2019 Stage 2 across different studies. Heterogeneity *P* is from Cochran’s Q test; Chr., chromosome; CI, confidence interval; OR, odds ratio;

**Supplementary Table 3. Summary results of 25 genetic variants identified in GWAS+GWAX2017 [3]**

| **SNP** | **Chr** | **Position (GRCh37)** | **Gene** | **EA/NEA** | **EAF** | **UK Biobank** | | **IGAP 2013 stage 1 + 2** | | **Meta-analysis** | | **Heterogeneity** | |
| --- | --- | --- | --- | --- | --- | --- | --- | --- | --- | --- | --- | --- | --- |
|  |  |  |  |  |  | **OR** | ***P* value** | **OR** | ***P* value** | **OR** | ***P* value** | ***I^2^*** | ***P* value** |
| rs6656401 | 1 | 207692049 | *CR1* | A/G | 0.178 | 1.13 | 2.87E-04 | 1.181399792 | 5.36E-24 | 1.17 | 1.16E-26 | 12% | 0.287 |
| rs6733839 | 2 | 127892810 | *BIN1* | T/C | 0.3738 | 1.12 | 1.22E-05 | 1.217135321 | 3.82E-44 | 1.19 | 3.09E-46 | 89% | 0.002 |
| rs35349669 | 2 | 234068476 | *INPP5D* | C/T | 0.5027 | 0.95 | 7.18E-02 | 0.927279731 | 2.83E-08 | 0.93 | 8.23E-09 | 0% | 0.364 |
| rs190982 | 5 | 88223420 | *MEF2C* | G/A | 0.3994 | 0.96 | 1.65E-01 | 0.926908893 | 3.02E-08 | 0.93 | 2.48E-08 | 35% | 0.215 |
| rs2074612 | 5 | 139714690 | *HBEGF* | T/C | 0.4379 | 1.07 | 1.25E-02 | 1.085130222 | 1.63E-07 | 1.08 | 8.00E-09 | 0% | 0.636 |
| rs9271087 | 6 | 32576170 | *MTCO3P1* | C/A | 0.2444 | 1.05 | 5.89E-02 | 1.110599545 | 2.36E-11 | 1.10 | 1.72E-11 | 65% | 0.091 |
| rs9272561 | 6 | 32607141 | *MTCO3P1* | G/A | 0.3277 | 1.05 | 9.28E-02 | 1.145681894 | 3.36E-09 | 1.11 | 1.65E-08 | 83% | 0.015 |
| rs10948363 | 6 | 47487762 | *CD2AP* | G/A | 0.2757 | 1.08 | 1.39E-02 | 1.100098807 | 4.73E-11 | 1.10 | 2.75E-12 | 0% | 0.490 |
| rs2718058 | 7 | 37841534 | *GPR141,EPDR1* | G/A | 0.3649 | 0.98 | 4.53E-01 | 0.925519572 | 4.53E-09 | 0.94 | 1.90E-08 | 70% | 0.067 |
| rs34995835 | 7 | 99990364 | *PILRA,ZCWPW1* | T/G | 0.3119 | 0.92 | 7.32E-03 | 0.913200333 | 8.51E-10 | 0.92 | 2.28E-11 | 0% | 0.708 |
| rs11771145 | 7 | 143110762 | *EPHA1* | A/G | 0.3489 | 0.98 | 4.57E-01 | 0.903029552 | 9.68E-14 | 0.92 | 2.24E-12 | 85% | 0.010 |
| rs28834970 | 8 | 27195121 | *PTK2B* | C/T | 0.3642 | 1.09 | 1.35E-03 | 1.104728938 | 6.95E-14 | 1.10 | 4.26E-16 | 0% | 0.706 |
| rs9331896 | 8 | 27467686 | *CLU* | C/T | 0.414 | 0.93 | 7.59E-03 | 0.864157703 | 3.99E-25 | 0.88 | 1.95E-25 | 83% | 0.017 |
| rs7920721 | 10 | 11720308 | *ECHDC3* | G/A | 0.3802 | 1.06 | 4.16E-02 | 1.071543358 | 3.08E-07 | 1.07 | 4.27E-08 | 0% | 0.651 |
| rs12292911 | 11 | 47449072 | *PSMC3* | A/G | 0.384 | 1.11 | 1.88E-04 | 1.076160915 | 1.64E-08 | 1.08 | 2.08E-11 | 0% | 0.354 |
| rs983392 | 11 | 59923508 | *MS4A6A* | G/A | 0.4013 | 0.93 | 6.81E-03 | 0.897537838 | 7.19E-16 | 0.90 | 3.57E-17 | 29% | 0.234 |
| rs10792832 | 11 | 85867875 | *FNTAL1* | A/G | 0.377 | 0.94 | 2.27E-02 | 0.869358235 | 6.53E-26 | 0.88 | 1.58E-25 | 86% | 0.008 |
| rs11218343 | 11 | 121435587 | *SORL1* | C/T | 0.03739 | 0.82 | 8.65E-03 | 0.769511024 | 9.08E-15 | 0.78 | 3.98E-16 | 0% | 0.392 |
| rs10498633 | 14 | 92926952 | *SLC24A4* | T/G | 0.2325 | 0.95 | 1.39E-01 | 0.909736756 | 5.24E-09 | 0.92 | 4.20E-09 | 43% | 0.185 |
| rs59685680 | 15 | 51001534 | *SPPL2A* | G/T | 0.1982 | 0.91 | 4.61E-03 | 0.920903524 | 4.30E-07 | 0.92 | 7.32E-09 | 0% | 0.713 |
| rs77493189 | 17 | 5118951 | *SCIMP* | G/T | 0.1227 | 1.16 | 2.62E-04 | 1.101309581 | 5.01E-07 | 1.11 | 9.60E-10 | 21% | 0.260 |
| rs3752231 | 19 | 1043638 | *ABCA7,CNN2* | T/C | 0.2551 | 1.07 | 2.13E-02 | 1.126032058 | 5.45E-11 | 1.11 | 8.85E-12 | 45% | 0.176 |
| rs4147929 | 19 | 1063443 | *ABCA7,HMHA1* | A/G | 0.1747 | 1.10 | 6.22E-03 | 1.153729802 | 9.46E-16 | 1.14 | 4.35E-17 | 32% | 0.227 |
| rs12459419 | 19 | 51728477 | *CD33* | T/C | 0.3277 | 0.94 | 2.75E-02 | 0.909827735 | 6.66E-08 | 0.92 | 8.58E-09 | 0% | 0.352 |
| rs7274581 | 20 | 55018260 | *CASS4* | C/T | 0.08461 | 0.93 | 1.21E-01 | 0.876078132 | 2.37E-08 | 0.89 | 1.22E-08 | 9% | 0.293 |

EA, effect allele; NEA, non-effect allele; EAF, effect allele frequency; *I^2^*, heterogeneity estimate; Heterogeneity *P* is from Cochran’s Q test; Chr., chromosome; CI, confidence interval; OR, odds ratio;

**Supplementary Table 4. Summary results of 26 genetic variants identified in GWAS+GWAX2018 [4]**

| **SNP** | **Chr** | **Position (GRCh37)** | **Meta *P*** | **EA** | **NEA** | **UK Biobank** | | | **IGAP 2013 stage 1 + 2** | | | **Heterogeneity** | |
| --- | --- | --- | --- | --- | --- | --- | --- | --- | --- | --- | --- | --- | --- |
|  |  |  |  |  |  | **BETA** | **SE** | ***P*** | **BETA** | **SE** | ***P*** | **I^2^** | ***P* value** |
| rs6656401 | 1 | 207692049 | 1.37E-29 | A | G | 0.0556 | 0.0098 | 1.41E-08 | 0.1667 | 0.0165 | 5.69E-24 | 97.02% | 7.07E-09 |
| rs6733839 | 2 | 127892810 | 2.37E-69 | T | C | 0.108 | 0.0099 | 1.06E-27 | 0.1965 | 0.0141 | 6.94E-44 | 96.21% | 2.79E-07 |
| rs35349669 | 2 | 234068476 | 3.58E-11 | T | C | 0.038 | 0.0101 | 1.61E-04 | 0.0755 | 0.0136 | 3.17E-08 | 79.59% | 2.69E-02 |
| rs9381040 | 6 | 41154650 | 1.55E-08 | T | C | -0.0295 | 0.01 | 3.21E-03 | -0.0705 | 0.0142 | 6.35E-07 | 82.06% | 1.82E-02 |
| rs9381563 | 6 | 47432637 | 5.83E-14 | T | C | -0.0408 | 0.01 | 4.66E-05 | -0.0879 | 0.0136 | 9.05E-11 | 87.15% | 5.27E-03 |
| rs1476679 | 7 | 100004446 | 9.93E-19 | T | C | 0.0631 | 0.01 | 2.66E-10 | 0.0891 | 0.0144 | 5.58E-10 | 54.53% | 1.38E-01 |
| rs10808026 | 7 | 143099133 | 1.13E-14 | A | C | -0.035 | 0.01 | 4.70E-04 | -0.1246 | 0.0169 | 1.52E-13 | 95.20% | 5.05E-06 |
| rs4236673 | 8 | 27464929 | 1.07E-28 | A | G | -0.0531 | 0.01 | 1.12E-07 | -0.1397 | 0.0136 | 9.21E-25 | 96.20% | 2.90E-07 |
| rs7920721 | 10 | 11720308 | 3.17E-11 | A | G | -0.0422 | 0.0099 | 2.20E-05 | -0.0691 | 0.0135 | 2.89E-07 | 61.27% | 1.08E-01 |
| rs12292911 | 11 | 47449072 | 3.30E-09 | A | G | 0.0257 | 0.01 | 1.03E-02 | 0.0734 | 0.013 | 1.88E-08 | 88.18% | 3.63E-03 |
| rs1582763 | 11 | 60021948 | 1.01E-18 | A | G | -0.0441 | 0.01 | 1.05E-05 | -0.1074 | 0.0135 | 1.81E-15 | 92.96% | 1.65E-04 |
| rs10792832 | 11 | 85867875 | 5.08E-36 | A | G | -0.0705 | 0.01 | 1.88E-12 | -0.14 | 0.0133 | 9.32E-26 | 94.27% | 2.96E-05 |
| rs11218343 | 11 | 121435587 | 4.58E-17 | T | C | 0.0413 | 0.0105 | 8.10E-05 | 0.262 | 0.0338 | 9.73E-15 | 97.43% | 4.50E-10 |
| rs17125924 | 14 | 53391680 | 1.34E-11 | A | G | -0.038 | 0.0099 | 1.28E-04 | -0.1258 | 0.0222 | 1.55E-08 | 92.34% | 3.04E-04 |
| rs12590654 | 14 | 92938855 | 8.21E-12 | A | G | -0.0431 | 0.01 | 1.67E-05 | -0.0965 | 0.0176 | 4.10E-08 | 85.63% | 8.34E-03 |
| rs59685680 | 15 | 51001534 | 9.17E-09 | T | G | 0.0298 | 0.01 | 2.87E-03 | 0.0824 | 0.0163 | 4.16E-07 | 86.78% | 5.95E-03 |
| rs593742 | 15 | 59045774 | 2.78E-11 | A | G | 0.0463 | 0.0101 | 4.57E-06 | 0.0681 | 0.0141 | 1.41E-06 | 36.70% | 2.09E-01 |
| rs889555 | 16 | 31122571 | 4.11E-08 | T | C | -0.0441 | 0.0102 | 1.59E-05 | -0.0511 | 0.0147 | 5.10E-04 | 0 | 6.96E-01 |
| rs4985556 | 16 | 70694000 | 3.67E-08 | A | C | 0.0286 | 0.0098 | 3.52E-03 | 0.1005 | 0.0208 | 1.41E-06 | 89.77% | 1.77E-03 |
| rs12444183 | 16 | 81773209 | 3.15E-08 | A | G | -0.0351 | 0.0101 | 5.00E-04 | -0.0569 | 0.0132 | 1.63E-05 | 41.87% | 1.90E-01 |
| rs7225151 | 17 | 5137047 | 6.06E-12 | A | G | 0.0456 | 0.0099 | 4.07E-06 | 0.0979 | 0.0192 | 3.66E-07 | 82.94% | 1.55E-02 |
| rs138190086 | 17 | 61538148 | 1.95E-09 | A | G | 0.0344 | 0.0098 | 4.74E-04 | 0.2942 | 0.0575 | 3.08E-07 | 94.96% | 8.43E-06 |
| rs3752231 | 19 | 1043638 | 4.37E-13 | T | C | 0.0384 | 0.01 | 1.17E-04 | 0.1187 | 0.0181 | 4.89E-11 | 93.37% | 1.03E-04 |
| rs41289512 | 19 | 45351516 | 6.70E-255 | C | G | -0.2244 | 0.0093 | 6.30E-130 | -1.6384 | 0.0594 | 2.24E-167 | 99.82% | 2.66E-122 |
| rs12459419 | 19 | 51728477 | 7.97E-09 | T | C | -0.0294 | 0.0099 | 3.08E-03 | -0.0945 | 0.0175 | 6.49E-08 | 90.46% | 1.20E-03 |
| rs6069736 | 20 | 54983075 | 2.00E-10 | T | C | -0.0391 | 0.0102 | 1.32E-04 | -0.1239 | 0.0241 | 2.83E-07 | 90.48% | 1.19E-03 |

EA, effect allele; NEA, non-effect allele; EAF, effect allele frequency; BETA, effect size for EA; SE, standard error; *I^2^*, heterogeneity estimate; Heterogeneity *P* is from Cochran’s Q test; Chr., chromosome; CI, confidence interval; OR, odds ratio;

**Supplementary Table 5. Summary results of 33 genetic variants identified in GWAS+GWAX2021a [5]**

| **SNP** | **Chr** | **Position (GRCh37)** | **EA** | **NEA** | **EAF** | **Meta-analysis** | | | **IGAP 2019 stage 1** | | | **UK Biobank** | | | **Heterogeneity** | |
| --- | --- | --- | --- | --- | --- | --- | --- | --- | --- | --- | --- | --- | --- | --- | --- | --- |
|  |  |  |  |  |  | **BETA** | **SE** | ***P* value** | **BETA** | **SE** | ***P* value** | **BETA** | **SE** | ***P* value** | ***I^2^*** | ***P* value** |
| rs4575098 | 1 | 161155392 | A | G | 0.23 | 0.061 | 0.011 | 4.30E-08 | 0.038 | 0.017 | 2.34E-02 | 0.079 | 0.015 | 2.50E-07 | 71% | 6.37E-02 |
| rs679515 | 1 | 207750568 | T | C | 0.18 | 0.123 | 0.012 | 1.40E-23 | 0.151 | 0.018 | 1.56E-16 | 0.100 | 0.017 | 1.50E-09 | 77% | 3.89E-02 |
| rs268134 | 2 | 65608363 | A | G | 0.25 | 0.062 | 0.011 | 1.54E-08 | 0.066 | 0.017 | 6.57E-05 | 0.059 | 0.015 | 4.50E-05 | 0% | 7.44E-01 |
| rs143080277 | 2 | 106366056 | T | C | 1.00 | -0.521 | 0.073 | 1.28E-12 | -0.458 | 0.106 | 1.51E-05 | -0.578 | 0.102 | 7.60E-09 | 0% | 4.11E-01 |
| rs6733839 | 2 | 127892810 | T | C | 0.39 | 0.156 | 0.010 | 1.10E-54 | 0.169 | 0.015 | 4.02E-28 | 0.146 | 0.013 | 4.60E-28 | 27% | 2.43E-01 |
| rs10933431 | 2 | 233981912 | C | G | 0.78 | 0.077 | 0.012 | 1.41E-10 | 0.100 | 0.019 | 2.55E-07 | 0.063 | 0.015 | 3.50E-05 | 56% | 1.30E-01 |
| rs4351014 | 4 | 11027619 | T | C | 0.26 | 0.071 | 0.011 | 2.59E-11 | 0.068 | 0.016 | 1.96E-05 | 0.073 | 0.014 | 2.10E-07 | 0% | 7.92E-01 |
| rs36096565 | 6 | 32560025 | A | G | 0.79 | 0.104 | 0.013 | 2.88E-15 | 0.097 | 0.023 | 1.91E-05 | 0.107 | 0.016 | 4.00E-11 | 0% | 7.39E-01 |
| rs187370608 | 6 | 40942196 | A | G | 0.00 | 0.797 | 0.080 | 1.83E-23 | 0.719 | 0.107 | 1.82E-11 | 0.895 | 0.120 | 7.60E-14 | 16% | 2.74E-01 |
| rs1385742 | 6 | 47595155 | A | T | 0.35 | 0.069 | 0.010 | 1.11E-11 | 0.088 | 0.016 | 2.23E-08 | 0.056 | 0.013 | 2.40E-05 | 59% | 1.21E-01 |
| rs1859788 | 7 | 99971834 | A | G | 0.32 | -0.090 | 0.010 | 3.28E-18 | -0.065 | 0.016 | 4.17E-05 | -0.107 | 0.014 | 9.40E-16 | 75% | 4.34E-02 |
| rs12703526 | 7 | 143107588 | T | G | 0.52 | 0.065 | 0.010 | 9.63E-12 | 0.064 | 0.015 | 1.15E-05 | 0.066 | 0.013 | 1.20E-07 | 0% | 9.19E-01 |
| rs867230 | 8 | 27468503 | A | C | 0.59 | 0.105 | 0.010 | 7.71E-26 | 0.133 | 0.016 | 3.49E-17 | 0.086 | 0.013 | 3.70E-11 | 81% | 2.12E-02 |
| rs7920721 | 10 | 11720308 | A | G | 0.62 | -0.067 | 0.010 | 1.08E-11 | -0.078 | 0.015 | 1.94E-07 | -0.058 | 0.013 | 6.70E-06 | 0% | 3.18E-01 |
| rs1171814 | 10 | 61645833 | T | G | 0.48 | -0.052 | 0.010 | 3.80E-08 | -0.035 | 0.014 | 1.59E-02 | -0.066 | 0.013 | 1.90E-07 | 63% | 9.94E-02 |
| rs1878036 | 10 | 82280137 | T | G | 0.21 | -0.070 | 0.012 | 2.74E-09 | -0.079 | 0.018 | 1.29E-05 | -0.064 | 0.016 | 2.40E-05 | 0% | 5.32E-01 |
| rs10437655 | 11 | 47391948 | A | G | 0.39 | 0.063 | 0.010 | 6.91E-11 | 0.084 | 0.015 | 1.22E-08 | 0.048 | 0.013 | 2.50E-04 | 70% | 6.76E-02 |
| rs72924626 | 11 | 60095740 | T | C | 0.63 | 0.089 | 0.010 | 9.33E-20 | 0.115 | 0.015 | 7.91E-15 | 0.069 | 0.013 | 2.60E-07 | 82% | 1.86E-02 |
| rs10792832 | 11 | 85867875 | A | G | 0.37 | -0.103 | 0.010 | 5.21E-26 | -0.120 | 0.015 | 7.56E-16 | -0.091 | 0.013 | 3.00E-12 | 53% | 1.44E-01 |
| rs11218343 | 11 | 121435587 | T | C | 0.96 | 0.186 | 0.025 | 5.59E-14 | 0.205 | 0.037 | 2.63E-08 | 0.171 | 0.033 | 2.90E-07 | 0% | 4.89E-01 |
| rs17125924 | 14 | 53391680 | A | G | 0.91 | -0.102 | 0.016 | 3.69E-10 | -0.122 | 0.025 | 6.62E-07 | -0.087 | 0.022 | 8.10E-05 | 14% | 2.82E-01 |
| rs12590654 | 14 | 92938855 | A | G | 0.34 | -0.076 | 0.010 | 7.45E-14 | -0.091 | 0.016 | 8.73E-09 | -0.066 | 0.013 | 1.30E-06 | 31% | 2.30E-01 |
| rs12592778 | 15 | 50992311 | A | G | 0.19 | -0.074 | 0.012 | 1.74E-09 | -0.075 | 0.019 | 5.24E-05 | -0.072 | 0.016 | 6.40E-06 | 0% | 9.06E-01 |
| rs442495 | 15 | 59022615 | T | C | 0.68 | 0.068 | 0.010 | 2.67E-11 | 0.069 | 0.015 | 5.44E-06 | 0.066 | 0.014 | 1.30E-06 | 0% | 8.74E-01 |
| rs117618017 | 15 | 63569902 | T | C | 0.14 | 0.085 | 0.015 | 1.05E-08 | 0.094 | 0.026 | 2.38E-04 | 0.080 | 0.018 | 1.70E-05 | 0% | 6.61E-01 |
| rs2884738 | 16 | 31126321 | A | C | 0.28 | -0.062 | 0.011 | 4.47E-09 | -0.045 | 0.016 | 4.96E-03 | -0.075 | 0.014 | 9.10E-08 | 48% | 1.65E-01 |
| rs61182333 | 17 | 5133128 | T | C | 0.12 | 0.085 | 0.014 | 1.35E-09 | 0.087 | 0.021 | 2.18E-05 | 0.083 | 0.019 | 7.20E-06 | 0% | 8.73E-01 |
| rs4311 | 17 | 61560763 | T | C | 0.47 | -0.054 | 0.010 | 1.21E-08 | -0.048 | 0.014 | 8.18E-04 | -0.059 | 0.013 | 5.10E-06 | 0% | 5.67E-01 |
| rs12151021 | 19 | 1050874 | A | G | 0.33 | 0.077 | 0.011 | 2.41E-13 | 0.107 | 0.017 | 2.56E-10 | 0.058 | 0.014 | 1.40E-05 | 80% | 2.39E-02 |
| rs429358 | 19 | 45411941 | T | C | 0.84 | -1.179 | 0.013 | 0 | -1.202 | 0.019 | 1.17e-881 | -1.160 | 0.017 | 2.4E-966 | 62% | 1.06E-01 |
| rs3865444 | 19 | 51727962 | A | C | 0.33 | -0.058 | 0.010 | 1.29E-08 | -0.080 | 0.016 | 3.93E-07 | -0.042 | 0.013 | 1.90E-03 | 71% | 6.53E-02 |
| rs6014724 | 20 | 54998544 | A | G | 0.91 | 0.109 | 0.017 | 1.07E-10 | 0.132 | 0.026 | 3.65E-07 | 0.093 | 0.022 | 5.80E-05 | 24% | 2.50E-01 |
| rs2830489 | 21 | 28148191 | T | C | 0.27 | -0.059 | 0.011 | 3.09E-08 | -0.084 | 0.016 | 2.42E-07 | -0.040 | 0.014 | 3.30E-03 | 76% | 4.27E-02 |

EA, effect allele; NEA, non-effect allele; EAF, effect allele frequency; BETA, effect size for EA; SE, standard error; *I^2^*, heterogeneity estimate; Heterogeneity *P* is from Cochran’s Q test; Chr., chromosome; CI, confidence interval; OR, odds ratio;

**Supplementary Table 6. Summary results of 36 genetic variants identified in GWAS+GWAX2021c [6]**

| **SNP** | **Gene** | **Chr** | **Position (GRCh37)** | **EA/NEA** | **GR@ACE** | | **IGAP** | | **UKB dataset** | | **Heterogeneity** | |
| --- | --- | --- | --- | --- | --- | --- | --- | --- | --- | --- | --- | --- |
|  |  |  |  |  | **OR[95%CI]** | ***P* value** | **OR[95%CI]** | ***P* value** | **OR[95%CI]** | ***P* value** | ***I^2^*** | ***P* value** |
| rs4844610 | *CR1* | 1 | 207802552 | A/C | 1.08[1.01-1.16] | 2.09E-02 | 1.17[1.13-1.21] | 3.60E-24 | 1.11[1.07-1.15] | 1.09E-08 | 69% | 3.99E-02 |
| rs2192939 | *PRKD3/NDUFAF7* | 2 | 37484726 | A/G | 1.05[1.00-1.11] | 4.34E-02 | 1.05[1.02-1.08] | 1.65E-03 | 1.06[1.03-1.09] | 3.26E-05 | 0% | 8.85E-01 |
| rs6733839 | *BIN1* | 2 | 127892810 | C/T | 0.86[0.81-0.90] | 1.07E-08 | 0.83[0.81-0.86] | 2.05E-44 | 0.85[0.83-0.88] | 1.05E-27 | 17% | 2.99E-01 |
| rs10933431 | *INPP5D* | 2 | 233981912 | C/G | 1.05[0.99-1.11] | 1.34E-01 | 1.10[1.06-1.13] | 3.42E-09 | 1.07[1.03-1.10] | 1.90E-04 | 2% | 3.59E-01 |
| rs4351014 | *HS3ST1* | 4 | 11027619 | C/T | 0.97[0.92-1.02] | 2.62E-01 | 0.93[0.91-0.96] | 1.96E-05 | 0.93[0.90-0.96] | 5.67E-06 | 8% | 3.37E-01 |
| rs9275152 | *HLA* | 6 | 32652196 | C/T | 0.90[0.81-1.00] | 4.67E-02 | 0.86[0.82-0.90] | 2.76E-11 | 0.89[0.85-0.94] | 1.11E-05 | 0% | 5.29E-01 |
| rs75932628 | *TREM2* | 6 | 41129252 | C/T | 0.49[0.30-0.82] | 6.76E-03 | 0.50[0.41-0.60] | 2.95E-12 | NA | NA | 0% | 9.40E-01 |
| rs9381040 | *TREML2* | 6 | 41154650 | C/T | 1.01[0.96-1.07] | 7.05E-01 | 1.07[1.04-1.10] | 6.22E-07 | 1.05[1.02-1.08] | 3.21E-03 | 48% | 1.46E-01 |
| rs9381564 | *CD2AP* | 6 | 47443806 | A/G | 0.92[0.87-0.97] | 3.30E-03 | 0.92[0.89-0.94] | 1.29E-10 | 0.94[0.91-0.97] | 1.06E-04 | 0% | 6.21E-01 |
| rs1859788 | *PILRA* | 7 | 99971834 | A/G | 0.92[0.87-0.97] | 2.75E-03 | 0.92[0.90-0.95] | 1.22E-09 | 0.91[0.88-0.94] | 2.42E-10 | 0% | 8.62E-01 |
| rs56402156 | *EPHA1* | 7 | 143103481 | A/G | 0.93[0.88-0.99] | 3.35E-02 | 0.90[0.88-0.93] | 1.46E-10 | 0.94[0.91-0.97] | 3.79E-04 | 60% | 7.96E-02 |
| rs73223431 | *PTK2B* | 8 | 27219987 | C/T | 0.98[0.93-1.03] | 3.44E-01 | 0.91[0.89-0.93] | 6.30E-14 | 0.93[0.91-0.96] | 3.88E-06 | 71% | 3.04E-02 |
| rs9331896 | *CLU* | 8 | 27467686 | C/T | 0.94[0.89-0.99] | 1.21E-02 | 0.88[0.85-0.90] | 4.63E-24 | 0.93[0.90-0.95] | 2.26E-07 | 70% | 3.67E-02 |
| rs34173062 | *SHARPIN* | 8 | 145158607 | A/G | 1.26[1.13-1.40] | 2.59E-05 | 1.09[1.01-1.19] | 3.58E-02 | 1.16[1.10-1.23] | 1.38E-07 | 57% | 9.59E-02 |
| rs7920721 | *ECHDC3* | 10 | 11720308 | A/G | 1.02[0.96-1.07] | 5.51E-01 | 0.93[0.90-0.95] | 2.31E-09 | 0.94[0.91-0.97] | 2.20E-05 | 72% | 2.85E-02 |
| rs3740688 | *SPI1* | 11 | 47380340 | T/G | 1.08[1.02-1.13] | 4.95E-03 | 1.09[1.07-1.12] | 5.46E-13 | 1.04[1.01-1.07] | 1.07E-02 | 72% | 2.90E-02 |
| rs1582763 | *MS4A4A* | 11 | 60021948 | A/G | 0.93[0.88-0.98] | 4.75E-03 | 0.89[0.87-0.91] | 2.36E-19 | 0.94[0.91-0.96] | 1.05E-05 | 75% | 1.76E-02 |
| rs3851179 | *PICALM* | 11 | 85868640 | C/T | 1.10[1.04-1.16] | 3.82E-04 | 1.14[1.11-1.17] | 6.03E-25 | 1.11[1.08-1.14] | 1.75E-12 | 18% | 2.96E-01 |
| rs11218343 | *SORL1* | 11 | 121435587 | C/T | 0.87[0.75-1.00] | 5.09E-02 | 0.80[0.75-0.85] | 2.88E-12 | 0.86[0.80-0.93] | 9.40E-05 | 22% | 2.78E-01 |
| rs17125924 | *FERMT2* | 14 | 53391680 | A/G | 0.88[0.79-0.97] | 1.25E-02 | 0.88[0.84-0.92] | 1.42E-09 | 0.91[0.87-0.96] | 1.27E-04 | 0% | 5.70E-01 |
| rs11623019 | *RIN3/SLC2A4* | 14 | 92936971 | C/T | 0.94[0.89-1.00] | 4.30E-02 | 0.92[0.90-0.95] | 1.13E-07 | 0.92[0.89-0.95] | 3.13E-06 | 0% | 7.65E-01 |
| rs593742 | *ADAM10* | 15 | 59045774 | A/G | 1.13[1.06-1.20] | 7.07E-05 | 1.07[1.05-1.10] | 1.25E-07 | 1.07[1.04-1.11] | 4.56E-06 | 24% | 2.68E-01 |
| rs117618017 | *APH1B* | 15 | 63569902 | C/T | 0.89[0.83-0.95] | 8.78E-04 | 0.91[0.87-0.96] | 2.38E-04 | 0.92[0.88-0.95] | 1.91E-05 | 0% | 7.34E-01 |
| rs7185636 | *IQCK* | 16 | 19808163 | C/T | 0.95[0.89-1.01] | 0.124 | 0.92[0.89-0.95] | 8.39E-08 | 1.00[0.97-1.04] | 8.55E-01 | 86% | 6.14E-04 |
| rs4985556 | *IL34* | 16 | 70694000 | A/C | 1.07[0.98-1.15] | 1.26E-01 | 1.10[1.05-1.14] | 3.82E-06 | 1.06[1.02-1.11] | 3.41E-03 | 0% | 4.82E-01 |
| rs12444183 | *PLCG2* | 16 | 81773209 | A/G | 0.96[0.91-1.02] | 1.69E-01 | 0.95[0.92-0.97] | 1.74E-05 | 0.95[0.92-0.98] | 5.00E-04 | 0% | 9.40E-01 |
| rs72835017 | *MINK1/CHRNE* | 17 | 4746294 | A/G | 0.81[0.74-0.89] | 9.49E-06 | 0.92[0.88-0.96] | 6.59E-05 | 0.93[0.89-0.98] | 4.41E-03 | 73% | 2.33E-02 |
| rs75511804 | *SCIMP* | 17 | 5138304 | C/T | 0.87[0.80-0.94] | 3.97E-04 | 0.92[0.89-0.96] | 1.07E-05 | 0.90[0.87-0.94] | 2.51E-06 | 0% | 3.95E-01 |
| rs386572859 | *MAPT/KANSL1* | 17 | 44368216 | A/G | 0.81[0.75-0.87] | 7.95E-09 | NA | NA | 0.99[0.95-1.03] | 5.13E-01 | 95% | < 0.0001 |
| rs4311 | *ACE* | 17 | 61560763 | C/T | 1.06[1.00-1.11] | 3.36E-02 | 1.05[1.02-1.08] | 8.18E-04 | 1.07[1.04-1.10] | 1.02E-06 | 0% | 6.45E-01 |
| rs3752231 | *ABCA7* | 19 | 1043638 | C/T | 0.93[0.88-0.98] | 1.10E-02 | 0.90[0.87-0.93] | 5.57E-12 | 0.94[0.91-0.97] | 1.17E-04 | 41% | 1.82E-01 |
| rs429358 | *APOE4* | 19 | 45411941 | C/T | 2.55[2.37-2.74] | 3.50E-136 | 3.33[3.20-3.45] | 0 | 2.85[2.76-2.94] | 0 | 96% | 1.33E-12 |
| rs7412 | *APOE2* | 19 | 45412079 | C/T | 1.51[1.34-1.69] | 5.90E-12 | 1.60[1.50-1.69] | 6.40E-53 | 1.59[1.51-1.68] | 9.01E-62 | 0% | 6.95E-01 |
| rs12459419 | *CD33* | 19 | 51728477 | C/T | 1.02[0.97-1.08] | 4.04E-01 | 1.08[1.05-1.12] | 4.51E-07 | 1.05[1.02-1.08] | 3.06E-03 | 54% | 1.14E-01 |
| rs6024870 | *CASS4* | 20 | 54997568 | A/G | 0.92[0.85-1.00] | 6.05E-02 | 0.88[0.85-0.92] | 3.46E-08 | 0.91[0.87-0.96] | 4.51E-04 | 0% | 3.87E-01 |
| rs2154481 | *APP* | 21 | 27473875 | C/T | 0.93[0.88-0.98] | 3.51E-03 | 0.95[0.93-0.97] | 2.10E-05 | 0.95[0.93-0.98] | 6.11E-04 | 0% | 7.67E-01 |

EA, effect allele; NEA, non-effect allele; EAF, effect allele frequency; *I^2^*, heterogeneity estimate; Heterogeneity *P* is from Cochran’s Q test; Chr., chromosome; CI, confidence interval; OR, odds ratio; NA, not available;

**Supplementary Table 7. Summary results of 83 genetic variants identified in GWAS+GWAX2022a [7]**

| **SNP** | **Chr** | **Position (cGRCh38)** | **Gene** | **EA/NEA** | **Stage I** | | **Stage II** | | **Stage I + II** | | **Heterogeneity** | |
| --- | --- | --- | --- | --- | --- | --- | --- | --- | --- | --- | --- | --- |
|  |  |  |  |  | **OR[95%CI]** | ***P* value** | **OR[95%CI]** | ***P* value** | **OR[95%CI]** | ***P* value** | ***I^2^*** | ***P* value** |
| rs141749679 | 1 | 109345810 | *SORT1* | C/T | 1.37 (1.19-1.57) | 7.99E-06 | 1.41 (1.17-1.7) | 2.36E-04 | 1.38 (1.24-1.54) | 7.54E-09 | 0% | 9.02E-01 |
| rs679515 | 1 | 207577223 | *CR1* | T/C | 1.13 (1.11-1.16) | 5.15E-33 | 1.12 (1.09-1.16) | 1.51E-14 | 1.13 (1.11-1.15) | 7.16E-46 | 27% | 1.63E-01 |
| rs72777026 | 2 | 9558882 | *ADAM17* | G/A | 1.06 (1.03-1.08) | 1.91E-06 | 1.06 (1.02-1.1) | 4.14E-03 | 1.06 (1.04-1.08) | 2.72E-08 | 0% | 8.12E-01 |
| rs17020490 | 2 | 37304796 | *PRKD3* | C/T | 1.06 (1.03-1.08) | 3.29E-06 | 1.06 (1.03-1.1) | 2.33E-04 | 1.06 (1.04-1.08) | 3.29E-09 | 4% | 4.09E-01 |
| rs143080277 | 2 | 105749599 | *NCK2* | C/T | 1.48 (1.31-1.66) | 7.89E-11 | 1.44 (1.17-1.78) | 6.29E-04 | 1.47 (1.33-1.63) | 2.07E-13 | 11% | 3.41E-01 |
| rs6733839 | 2 | 127135234 | *BIN1* | T/C | 1.18 (1.16-1.2) | 6.48E-90 | 1.16 (1.13-1.18) | 2.06E-30 | 1.17 (1.16-1.19) | 6.06E-118 | 57% | 3.56E-03 |
| rs139643391 | + | 202878716 | *WDR12* | T/TC | 0.94 (0.92-0.96) | 2.56E-07 | 0.93 (0.88-0.99) | 1.29E-02 | 0.94 (0.92-0.96) | 1.08E-08 | 49% | 3.54E-02 |
| rs10933431 | 2 | 233117202 | *INPP5D* | G/C | 0.92 (0.9-0.94) | 1.04E-17 | 0.96 (0.93-0.99) | 4.70E-03 | 0.93 (0.92-0.95) | 3.62E-18 | 18% | 2.54E-01 |
| rs16824536 | 3 | 155069722 | *MME* | A/G | 0.92 (0.89-0.95) | 3.78E-06 | 0.92 (0.87-0.97) | 2.75E-03 | 0.92 (0.89-0.95) | 3.63E-08 | 0% | 5.78E-01 |
| rs61762319 | 3 | 155084189 | *MME* | G/A | 1.15 (1.1-1.21) | 2.14E-08 | 1.18 (1.08-1.29) | 2.14E-04 | 1.16 (1.11-1.21) | 2.16E-11 | 11% | 3.34E-01 |
| rs3822030 | 4 | 993555 | *IDUA* | G/T | 0.95 (0.93-0.97) | 5.04E-10 | 0.96 (0.93-0.99) | 4.31E-03 | 0.95 (0.94-0.96) | 8.29E-12 | 3% | 4.20E-01 |
| rs6846529 | 4 | 11023507 | *CLNK* | C/T | 1.07 (1.05-1.09) | 1.25E-13 | 1.06 (1.03-1.09) | 3.16E-05 | 1.07 (1.05-1.08) | 2.20E-17 | 41% | 4.77E-02 |
| rs2245466 | 4 | 40197226 | *RHOH* | G/C | 1.05 (1.03-1.07) | 3.05E-07 | 1.05 (1.02-1.07) | 1.05E-03 | 1.05 (1.03-1.06) | 1.22E-09 | 14% | 2.94E-01 |
| rs112403360 | 5 | 14724304 | *ANKH* | A/T | 1.08 (1.04-1.11) | 3.42E-06 | 1.14 (1.07-1.21) | 4.63E-05 | 1.09 (1.06-1.12) | 2.27E-09 | 50% | 1.59E-02 |
| rs62374257 | 5 | 86927378 | *COX7C* | C/T | 1.07 (1.05-1.09) | 1.41E-13 | 1.06 (1.02-1.1) | 1.96E-03 | 1.07 (1.05-1.09) | 1.38E-15 | 43% | 4.52E-02 |
| rs871269 | 5 | 151052827 | *TNIP1* | T/C | 0.96 (0.94-0.98) | 3.42E-06 | 0.96 (0.93-0.98) | 6.58E-04 | 0.96 (0.95-0.97) | 8.67E-09 | 52% | 9.63E-03 |
| rs113706587 | 5 | 180201150 | *RASGEF1C* | A/G | 1.1 (1.07-1.13) | 3.38E-12 | 1.09 (1.05-1.13) | 1.30E-05 | 1.09 (1.07-1.12) | 2.22E-16 | 0% | 8.63E-01 |
| rs6605556 | 6 | 32615322 | *HLA-DQA1* | G/A | 0.91 (0.89-0.93) | 1.05E-17 | 0.94 (0.9-0.97) | 5.83E-04 | 0.91 (0.9-0.93) | 7.07E-20 | 7% | 3.81E-01 |
| rs10947943 | 6 | 41036354 | *UNC5CL* | A/G | 0.95 (0.93-0.97) | 6.21E-06 | 0.93 (0.9-0.96) | 2.98E-05 | 0.94 (0.93-0.96) | 1.13E-09 | 4% | 4.12E-01 |
| rs143332484 | 6 | 41161469 | *TREM2* | T/C | 1.4 (1.3-1.5) | 6.04E-19 | 1.44 (1.26-1.65) | 7.08E-08 | 1.41 (1.32-1.5) | 2.78E-25 | 59% | 2.40E-03 |
| rs75932628 | 6 | 41161514 | *TREM2* | T/C | 2.42 (2.06-2.84) | 1.39E-27 | 2.3 (1.8-2.94) | 2.34E-11 | 2.39 (2.09-2.73) | 2.53E-37 | 0% | 6.90E-01 |
| rs60755019 | 6 | 41181270 | *TREML2* | G/A | 1.55 (1.32-1.83) | 1.78E-07 | 1.51 (1.02-2.25) | 4.09E-02 | 1.55 (1.33-1.8) | 2.07E-08 | 50% | 7.58E-02 |
| rs7767350 | 6 | 47517390 | *CD2AP* | T/C | 1.06 (1.05-1.08) | 5.05E-12 | 1.1 (1.07-1.13) | 2.95E-12 | 1.08 (1.06-1.09) | 7.94E-22 | 0% | 8.70E-01 |
| rs785129 | 6 | 114291731 | *HS3ST5* | T/C | 1.05 (1.03-1.06) | 2.94E-07 | 1.04 (1.01-1.07) | 2.11E-03 | 1.04 (1.03-1.06) | 2.40E-09 | 4% | 4.10E-01 |
| rs6943429 | 7 | 7817263 | *UMAD1* | T/C | 1.04 (1.03-1.06) | 2.98E-07 | 1.05 (1.03-1.08) | 7.46E-05 | 1.05 (1.03-1.06) | 1.03E-10 | 47% | 2.44E-02 |
| rs10952097 | 7 | 8204382 | *ICA1* | T/C | 1.07 (1.04-1.1) | 3.28E-07 | 1.07 (1.02-1.12) | 5.95E-03 | 1.07 (1.05-1.1) | 6.81E-09 | 51% | 1.51E-02 |
| rs13237518 | 7 | 12229967 | *TMEM106B* | A/C | 0.96 (0.94-0.98) | 5.13E-07 | 0.95 (0.93-0.97) | 1.58E-05 | 0.96 (0.94-0.97) | 4.88E-11 | 47% | 2.28E-02 |
| rs1160871 | 7 | 28129126 | *JAZF1* | G/GTCTT | 0.95 (0.93-0.97) | 1.12E-07 | 0.95 (0.91-1) | 2.96E-02 | 0.95 (0.93-0.97) | 9.83E-09 | 0% | 8.84E-01 |
| rs6966331 | 7 | 37844191 | *EPDR1* | T/C | 0.96 (0.95-0.98) | 4.81E-06 | 0.95 (0.92-0.97) | 1.36E-05 | 0.96 (0.94-0.97) | 4.64E-10 | 0% | 5.09E-01 |
| rs76928645 | 7 | 54873635 | *SEC61G* | T/C | 0.93 (0.9-0.95) | 1.51E-08 | 0.94 (0.91-0.98) | 2.32E-03 | 0.93 (0.91-0.95) | 1.62E-10 | 16% | 2.79E-01 |
| rs7384878 | 7 | 100334426 | *SPDYE3* | C/T | 0.93 (0.91-0.94) | 2.13E-18 | 0.92 (0.9-0.95) | 7.41E-10 | 0.92 (0.91-0.94) | 1.06E-26 | 0% | 6.53E-01 |
| rs11771145 | 7 | 143413669 | *EPHA1* | A/G | 0.94 (0.93-0.96) | 1.29E-12 | 0.96 (0.94-0.99) | 2.29E-03 | 0.95 (0.93-0.96) | 3.30E-14 | 53% | 9.94E-03 |
| rs1065712 | 8 | 11844613 | *CTSB* | C/G | 1.11 (1.07-1.16) | 5.46E-09 | 1.06 (1.01-1.11) | 2.53E-02 | 1.09 (1.06-1.12) | 1.94E-09 | 31% | 1.27E-01 |
| rs73223431 | 8 | 27362470 | *PTK2B* | T/C | 1.07 (1.05-1.09) | 5.34E-15 | 1.07 (1.05-1.1) | 1.17E-08 | 1.07 (1.06-1.08) | 4.03E-22 | 0% | 6.69E-01 |
| rs11787077 | 8 | 27607795 | *CLU* | T/C | 0.9 (0.89-0.92) | 2.43E-33 | 0.92 (0.89-0.94) | 6.60E-13 | 0.91 (0.9-0.92) | 1.70E-44 | 23% | 2.04E-01 |
| rs34173062 | 8 | 144103704 | *SHARPIN* | A/G | 1.12 (1.09-1.16) | 2.93E-12 | 1.14 (1.08-1.21) | 1.02E-05 | 1.13 (1.09-1.16) | 1.72E-16 | 40% | 5.99E-02 |
| rs1800978 | 9 | 104903697 | *ABCA1* | G/C | 1.07 (1.05-1.1) | 1.61E-08 | 1.04 (1.01-1.08) | 1.65E-02 | 1.06 (1.04-1.08) | 1.59E-09 | 25% | 1.82E-01 |
| rs7912495 | 10 | 11676714 | *USP6NL* | G/A | 1.06 (1.04-1.08) | 2.87E-12 | 1.07 (1.04-1.09) | 5.02E-08 | 1.06 (1.05-1.08) | 9.74E-19 | 13% | 3.03E-01 |
| rs7068231 | 10 | 60025170 | *ANK3* | T/G | 0.95 (0.94-0.97) | 6.79E-09 | 0.95 (0.92-0.97) | 9.67E-06 | 0.95 (0.94-0.96) | 3.32E-13 | 0% | 5.30E-01 |
| rs6586028 | 10 | 80494228 | *TSPAN14* | C/T | 0.92 (0.91-0.94) | 1.33E-14 | 0.93 (0.91-0.96) | 2.51E-06 | 0.93 (0.91-0.94) | 1.97E-19 | 0% | 5.44E-01 |
| rs6584063 | 10 | 96266650 | *BLNK* | G/A | 0.89 (0.86-0.93) | 1.84E-07 | 0.88 (0.83-0.94) | 8.26E-05 | 0.89 (0.86-0.92) | 6.73E-11 | 0% | 9.35E-01 |
| rs7908662 | 10 | 122413396 | *PLEKHA1* | G/A | 0.96 (0.95-0.98) | 3.30E-06 | 0.96 (0.93-0.98) | 1.71E-04 | 0.96 (0.95-0.97) | 2.59E-09 | 0% | 4.87E-01 |
| rs10437655 | 11 | 47370397 | *SPI1* | A/G | 1.06 (1.04-1.08) | 8.21E-12 | 1.05 (1.02-1.08) | 1.52E-03 | 1.06 (1.04-1.07) | 5.28E-14 | 13% | 3.13E-01 |
| rs1582763 | 11 | 60254475 | *MS4A4A* | A/G | 0.92 (0.9-0.93) | 1.65E-24 | 0.89 (0.87-0.91) | 2.87E-20 | 0.91 (0.9-0.92) | 3.74E-42 | 34% | 1.01E-01 |
| rs3851179 | 11 | 86157598 | *EED* | T/C | 0.9 (0.89-0.91) | 6.50E-36 | 0.91 (0.89-0.93) | 4.29E-14 | 0.9 (0.89-0.92) | 2.95E-48 | 38% | 6.59E-02 |
| rs74685827 | 11 | 121482368 | *SORL1* | G/T | 1.22 (1.15-1.29) | 8.63E-11 | 1.11 (1.01-1.23) | 3.65E-02 | 1.19 (1.13-1.25) | 2.81E-11 | 0% | 7.32E-01 |
| rs11218343 | 11 | 121564878 | *SORL1* | C/T | 0.85 (0.81-0.88) | 1.01E-14 | 0.82 (0.77-0.88) | 1.76E-08 | 0.84 (0.81-0.87) | 1.40E-21 | 3% | 4.19E-01 |
| rs6489896 | 12 | 113281983 | *TPCN1* | C/T | 1.08 (1.04-1.11) | 2.54E-06 | 1.08 (1.04-1.12) | 1.78E-04 | 1.08 (1.05-1.1) | 1.80E-09 | 28% | 1.47E-01 |
| rs17125924 | 14 | 52924962 | *FERMT2* | G/A | 1.09 (1.06-1.12) | 5.82E-10 | 1.11 (1.07-1.16) | 2.07E-07 | 1.1 (1.07-1.12) | 8.32E-16 | 0% | 9.15E-01 |
| rs7401792 | 14 | 92464917 | *SLC24A4* | G/A | 1.04 (1.02-1.06) | 7.15E-06 | 1.04 (1.01-1.07) | 1.91E-03 | 1.04 (1.02-1.05) | 4.83E-08 | 0% | 9.13E-01 |
| rs12590654 | 14 | 92472511 | *SLC24A4* | A/G | 0.93 (0.92-0.95) | 2.08E-15 | 0.94 (0.91-0.96) | 3.55E-07 | 0.93 (0.92-0.95) | 4.25E-21 | 0% | 7.49E-01 |
| rs7157106 | 14 | 105761758 | *IGH gene cluster* | A/G | 1.06 (1.04-1.08) | 1.46E-07 | 1.04 (1-1.08) | 3.49E-02 | 1.05 (1.03-1.07) | 1.99E-08 | 0% | 5.37E-01 |
| rs10131280 | 14 | 106665591 | *IGH gene cluster* | A/G | 0.94 (0.91-0.96) | 1.98E-07 | 0.94 (0.91-0.97) | 5.42E-04 | 0.94 (0.92-0.96) | 4.26E-10 | 0% | 9.77E-01 |
| rs8025980 | 15 | 50701814 | *SPPL2A* | G/A | 0.96 (0.95-0.98) | 6.09E-06 | 0.94 (0.91-0.97) | 2.96E-04 | 0.96 (0.94-0.97) | 1.32E-08 | 19% | 2.44E-01 |
| rs602602 | 15 | 58764824 | *MINDY2* | A/T | 0.94 (0.92-0.96) | 9.65E-12 | 0.95 (0.92-0.97) | 3.91E-05 | 0.94 (0.93-0.96) | 2.07E-15 | 18% | 2.53E-01 |
| rs117618017 | 15 | 63277703 | *APH1B* | T/C | 1.12 (1.09-1.15) | 1.75E-21 | 1.09 (1.05-1.13) | 1.21E-05 | 1.11 (1.09-1.13) | 2.15E-25 | 0% | 5.04E-01 |
| rs3848143 | 15 | 64131307 | *SNX1* | G/A | 1.05 (1.03-1.07) | 1.10E-06 | 1.07 (1.04-1.1) | 1.11E-05 | 1.05 (1.04-1.07) | 8.41E-11 | 0% | 6.15E-01 |
| rs12592898 | 15 | 78936857 | *CTSH* | A/G | 0.94 (0.92-0.97) | 1.28E-06 | 0.94 (0.91-0.98) | 8.69E-04 | 0.94 (0.92-0.96) | 4.18E-09 | 0% | 4.77E-01 |
| rs1140239 | 16 | 30010081 | *DOC2A* | T/C | 0.94 (0.93-0.96) | 4.61E-12 | 0.96 (0.92-0.99) | 1.37E-02 | 0.94 (0.93-0.96) | 2.59E-13 | 0% | 8.45E-01 |
| rs889555 | 16 | 31111250 | *BCKDK* | T/C | 0.95 (0.93-0.96) | 1.03E-09 | 0.96 (0.94-0.99) | 3.06E-03 | 0.95 (0.94-0.97) | 1.96E-11 | 1% | 4.35E-01 |
| rs4985556 | 16 | 70660097 | *IL34* | A/C | 1.06 (1.03-1.09) | 5.65E-06 | 1.09 (1.05-1.13) | 1.21E-05 | 1.07 (1.05-1.09) | 5.98E-10 | 24% | 1.90E-01 |
| rs450674 | 16 | 79574511 | *MAF* | C/T | 0.96 (0.94-0.97) | 1.07E-07 | 0.98 (0.95-1) | 4.31E-02 | 0.96 (0.95-0.98) | 3.16E-08 | 19% | 2.45E-01 |
| rs12446759 | 16 | 81739398 | *PLCG2* | G/A | 0.94 (0.93-0.96) | 3.60E-12 | 0.96 (0.94-0.99) | 3.41E-03 | 0.95 (0.94-0.96) | 1.22E-13 | 37% | 7.48E-02 |
| rs72824905 | 16 | 81908423 | *PLCG2* | G/C | 0.74 (0.67-0.81) | 2.66E-10 | 0.78 (0.64-0.94) | 8.15E-03 | 0.74 (0.68-0.81) | 8.48E-12 | 0% | 6.21E-01 |
| rs16941239 | 16 | 86420604 | *FOXF1* | A/T | 1.13 (1.07-1.19) | 2.93E-06 | 1.12 (1.05-1.21) | 1.20E-03 | 1.13 (1.08-1.17) | 1.29E-08 | 0% | 6.47E-01 |
| rs56407236 | 16 | 90103687 | *PRDM7* | A/G | 1.12 (1.08-1.15) | 1.28E-11 | 1.11 (1.05-1.17) | 1.09E-04 | 1.11 (1.08-1.14) | 6.47E-15 | 0% | 6.76E-01 |
| rs35048651 | 17 | 1728046 | *WDR81* | T/TGAG | 1.05 (1.03-1.07) | 4.79E-07 | 1.11 (1.06-1.16) | 4.11E-06 | 1.06 (1.04-1.08) | 7.67E-11 | 0% | 4.43E-01 |
| rs7225151 | 17 | 5233752 | *SCIMP* | A/G | 1.09 (1.06-1.12) | 2.60E-12 | 1.05 (1.01-1.08) | 7.75E-03 | 1.08 (1.05-1.1) | 4.13E-13 | 30% | 1.30E-01 |
| rs2242595 | 17 | 18156140 | *MYO15A* | A/G | 0.94 (0.92-0.97) | 4.55E-06 | 0.93 (0.9-0.96) | 5.16E-05 | 0.94 (0.92-0.96) | 1.11E-09 | 0% | 9.96E-01 |
| rs5848 | 17 | 44352876 | *GRN* | T/C | 1.07 (1.05-1.09) | 1.76E-12 | 1.08 (1.06-1.11) | 1.46E-09 | 1.07 (1.06-1.09) | 2.38E-20 | 13% | 3.10E-01 |
| rs199515 | 17 | 46779275 | *WNT3* | G/C | 0.94 (0.93-0.96) | 5.97E-09 | 0.93 (0.9-0.96) | 2.78E-05 | 0.94 (0.93-0.96) | 9.34E-13 | 59% | 2.36E-03 |
| rs616338 | 17 | 49219935 | *ABI3* | T/C | 1.34 (1.24-1.45) | 5.38E-13 | 1.23 (1.05-1.44) | 9.37E-03 | 1.32 (1.23-1.42) | 2.82E-14 | 28% | 1.54E-01 |
| rs2526377 | 17 | 58332680 | *TSPOAP1* | G/A | 0.96 (0.94-0.97) | 4.11E-08 | 0.95 (0.92-0.97) | 6.71E-06 | 0.95 (0.94-0.97) | 1.58E-12 | 54% | 6.98E-03 |
| rs4277405 | 17 | 63471557 | *ACE* | C/T | 0.93 (0.92-0.95) | 7.24E-16 | 0.95 (0.92-0.97) | 1.60E-05 | 0.94 (0.93-0.95) | 8.80E-20 | 0% | 8.86E-01 |
| rs12151021 | 19 | 1050875 | *ABCA7* | A/G | 1.11 (1.09-1.13) | 4.09E-30 | 1.09 (1.06-1.12) | 2.36E-09 | 1.1 (1.09-1.12) | 1.59E-37 | 47% | 2.87E-02 |
| rs149080927 | 19 | 1854254 | *KLF16* | G/GC | 1.05 (1.04-1.07) | 6.14E-09 | 1.04 (1.01-1.08) | 2.30E-02 | 1.05 (1.04-1.07) | 5.09E-10 | 0% | 6.43E-01 |
| rs9304690 | 19 | 49950060 | *SIGLEC11* | T/C | 1.05 (1.03-1.07) | 6.36E-06 | 1.06 (1.03-1.09) | 1.66E-04 | 1.05 (1.03-1.07) | 4.74E-09 | 13% | 3.06E-01 |
| rs587709 | 19 | 54267597 | *LILRB2* | C/T | 1.06 (1.04-1.08) | 3.88E-08 | 1.05 (1.02-1.08) | 2.21E-04 | 1.05 (1.04-1.07) | 3.63E-11 | 43% | 4.48E-02 |
| rs1358782 | 20 | 413334 | *RBCK1* | A/G | 0.95 (0.94-0.97) | 1.86E-06 | 0.96 (0.93-0.98) | 2.36E-03 | 0.95 (0.94-0.97) | 1.55E-08 | 35% | 9.96E-02 |
| rs6014724 | 20 | 56423488 | *CASS4* | G/A | 0.89 (0.86-0.91) | 4.84E-16 | 0.89 (0.85-0.94) | 1.56E-06 | 0.89 (0.87-0.91) | 4.13E-21 | 0% | 6.31E-01 |
| rs6742 | 20 | 63743088 | *SLC2A4RG* | T/C | 0.95 (0.93-0.97) | 2.49E-06 | 0.94 (0.91-0.97) | 2.22E-04 | 0.95 (0.93-0.97) | 2.58E-09 | 0% | 9.77E-01 |
| rs2154481 | 21 | 26101558 | *APP* | C/T | 0.95 (0.94-0.97) | 1.02E-09 | 0.96 (0.93-0.98) | 2.15E-04 | 0.95 (0.94-0.97) | 1.00E-12 | 0% | 4.73E-01 |
| rs2830489 | 21 | 26775872 | *ADAMTS1* | T/C | 0.95 (0.93-0.96) | 1.72E-09 | 0.97 (0.94-0.99) | 1.28E-02 | 0.95 (0.94-0.97) | 1.69E-10 | 28% | 1.52E-01 |

EA, effect allele; NEA, non-effect allele; EAF, effect allele frequency; *I^2^*, heterogeneity estimate; *I^2^* is calculated across different studies in Stage 1 and Stage 2. Heterogeneity *P* is from Cochran’s Q test; Chr., chromosome; CI, confidence interval; OR, odds ratio;

**References**

1. Lambert JC, Ibrahim-Verbaas CA, Harold D, Naj AC, Sims R, Bellenguez C, DeStafano AL, Bis JC, Beecham GW, Grenier-Boley B, et al: **Meta-analysis of 74,046 individuals identifies 11 new susceptibility loci for Alzheimer's disease.** *Nat Genet* 2013, **45:**1452-1458.

2. Kunkle BW, Grenier-Boley B, Sims R, Bis JC, Damotte V, Naj AC, Boland A, Vronskaya M, van der Lee SJ, Amlie-Wolf A, et al: **Genetic meta-analysis of diagnosed Alzheimer's disease identifies new risk loci and implicates Abeta, tau, immunity and lipid processing.** *Nat Genet* 2019, **51:**414-430.

3. Liu JZ, Erlich Y, Pickrell JK: **Case-control association mapping by proxy using family history of disease.** *Nat Genet* 2017, **49:**325-331.

4. Marioni RE, Harris SE, Zhang Q, McRae AF, Hagenaars SP, Hill WD, Davies G, Ritchie CW, Gale CR, Starr JM, et al: **GWAS on family history of Alzheimer's disease.** *Transl Psychiatry* 2018, **8:**99.

5. Schwartzentruber J, Cooper S, Liu JZ, Barrio-Hernandez I, Bello E, Kumasaka N, Young AMH, Franklin RJM, Johnson T, Estrada K, et al: **Genome-wide meta-analysis, fine-mapping and integrative prioritization implicate new Alzheimer's disease risk genes.** *Nat Genet* 2021, **53:**392-402.

6. de Rojas I, Moreno-Grau S, Tesi N, Grenier-Boley B, Andrade V, Jansen IE, Pedersen NL, Stringa N, Zettergren A, Hernandez I, et al: **Common variants in Alzheimer's disease and risk stratification by polygenic risk scores.** *Nat Commun* 2021, **12:**3417.

7. Bellenguez C, Kucukali F, Jansen IE, Kleineidam L, Moreno-Grau S, Amin N, Naj AC, Campos-Martin R, Grenier-Boley B, Andrade V, et al: **New insights into the genetic etiology of Alzheimer's disease and related dementias.** *Nat Genet* 2022, **54:**412-436.
